# Advancements in coupled processes numerical models: Upscaling aperture fields using spatial continuity

**DOI:** 10.1016/j.isci.2024.111094

**Published:** 2024-10-02

**Authors:** Gonçalo Benitez Cunha, Christopher Ian McDermott, Alexander Bond, Andrew Fraser-Harris, Roberto Emanuele Rizzo

**Affiliations:** 1 School of Geosciences, The University of Edinburgh, The King’s Buildings, James Hutton Road, Edinburgh EH9 3FE, UK; 2 INTERA, INTERA Geosciences UK Limited, 297 Euston Road, London NW1 3AD, UK; 3 Quintessa, 401 Faraday Street, Birchwood Park, Warrington WA3 6GA, UK; 4 Department of Earth Sciences, Utrecht University, Princetonlaan 4 3584 CB Utrecht, the Netherlands

**Keywords:** Earth sciences, Geology, Methods in earth sciences, Petrophysics

## Abstract

Fluid flow through fractured geological media is crucial in addressing the challenges posed by climate change, resource management, and energy exploration. Numerical models commonly employ fracture surface representations and aperture distribution models to simulate these processes. However, conventional statistical approaches often overlook the inherent spatial continuity and directionality within fracture data, impacting the accuracy of aperture geometry and subsequent flow simulations. This study investigates the benefits of incorporating spatial continuity information, derived from semi-variogram analysis, into numerical models. A Freiberg gneiss fracture aperture field was upscaled using both spatial continuity-informed and traditional arithmetic averaging methods. The comparative analysis reveals that incorporating spatial continuity during the upscaling process yields notable improvements in the accuracy of flow simulations, particularly when employing coarser mesh resolutions. This approach presents a promising alternative for enhancing the representation of fracture and aperture fields in numerical modeling across diverse applications, promoting a deeper understanding of fluid flow behavior in complex geological systems.

## Highlights

• A spatial continuity-based upscaling method capturing directional correlations
• Characterization of rock fracture aperture fields using variogram functions
• Spatial continuity upscaling includes sub-grid information, enhancing accuracy
• This method improves simulation results at coarser mesh resolutions

## Graphical abstract

**Figure:**
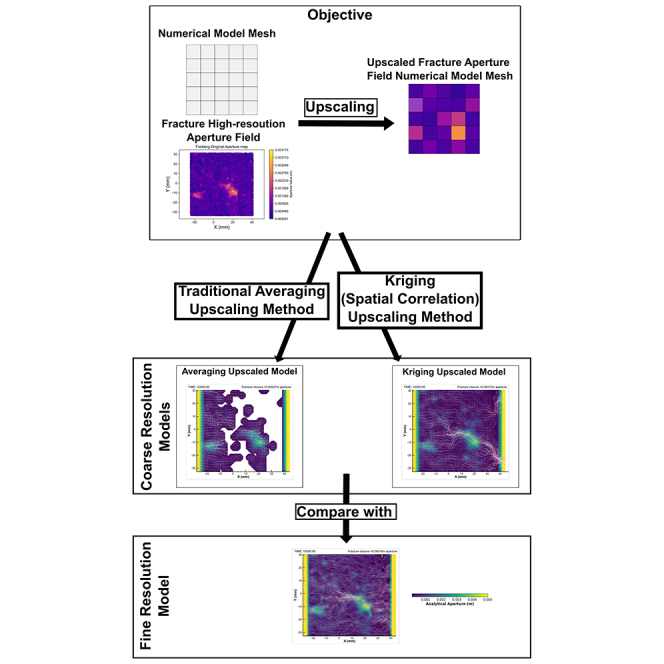

## Introduction

Climate change has underscored the urgency of exploring alternative energy sources and developing strategies for capturing and storing greenhouse gases. These initiatives present a series of scientific and engineering challenges, many of which hinge on our understanding of fluid flow through fractured geological media.^1^ Such processes are pivotal in a variety of contexts, including the management of hydrological resources,^2^ the extraction of geothermal energy,^3^^,^^4^^,^^5^ the storage of energy in forms such as hydrogen^6^ or natural gas, the disposal of nuclear waste,^7^^,^^8^^,^^9^^,^^10^ and the sequestration of greenhouse gases such as CO_2_.^11^^,^^12^ Open fractures in rocks are widely recognized for their role in enhancing fluid flow, particularly in relatively impermeable media. Studies have shown that the physical properties of rocks are of crucial importance for coupled processes numerical simulations since they dictate their behavior when subjected to physical inputs, such as temperature, stress, or fluid pressure.^13^^,^^14^^,^^15^^,^^16^^,^^17^^,^^18^^,^^19^ When simulating fluid flow through fractures, the fracture permeability is mainly influenced by the aperture distribution which, in turn, is a function of the roughness and the alignment of the fracture surfaces.^14^^,^^20^^,^^21^^,^^22^^,^^23^ Although the characterization of aperture distribution has seen limited detailed investigation, efforts have been made to quantify single fracture roughness. Summarized by^24^^,^^25^ existing methods range from the joint roughness coefficient (JRC),^26^^,^^27^^,^^28^ Fourier transforms and power spectra,^25^^,^^29^^,^^30^^,^^31^ fractal dimensions^25^^,^^32^ and probability density functions.^25^ However, these methods largely overlooked the directionality and the two-dimensional (2D) spatial continuity of the roughness, limiting their ability to accurately model fracture behavior in all directions. Recognizing this gap^33^ introduced the concept of semi-variogram for describing fracture roughness, though without providing a practical application to this concept. At the same time,^14^ introduced and applied the concept of spatial continuity to the impact of shear displacement on fracture aperture and permeability. Building on these foundations, more recently^34^^,^^35^ further developed the spatial continuity concept for modeling fracture permeability fields. However, the authors upscale the aperture distribution and only then calculate the permeability tensor. The anisotropy and angle are interpreted from the permeability tensor, not from a statistical spatial continuity analysis.

In response to these challenges, we present an original approach for characterizing a fracture aperture field through spatial continuity techniques. Specifically, we leverage the spatial continuity of a Freiberg gneiss single rough fracture aperture field, employing a kriging algorithm for upscaling in coupled thermal-hydraulic-mechanical-chemical (THMC) processes numerical simulations. Our method contrasts with traditional averaging techniques by accounting for spatial data continuity, offering a more accurate representation of fracture roughness to be used in numerical models. We test this method by comparing arithmetic averaging and ordinary kriging in finite element method (FEM) simulations of varying mesh sizes within a coupled hydro-mechanical model.

The objective of this study is to utilize spatial continuity information to guide a kriging algorithm, aiming to enhance the prediction of upscaled values in elements of THMC coupled processes numerical models. By comparing fine and coarse models using both aperture averaging and kriging, we assess the effectiveness of our technique by capturing the spatial continuity of the aperture field.

This approach represents a significant advancement in the modeling of fluid flow through fractured geological media, offering a more precise and reliable method for predicting the behavior of fractures in various environmental and energy-related applications.

## Spatial continuity theory

Given the limited application of semi-variograms in fracture flow studies, we begin by establishing the fundamental concepts that form the foundation of our proposed methodology. Spatial variables represent the cornerstone of spatial continuity theory. These variables, which characterize the physical environment, are defined by their variation across space due to underlying complex processes.^36^ Although their distribution may seem random, it is regarded as such due to the culmination of these poorly defined complex processes.^37^ A closer examination may reveal an inherent structure or pattern at certain scales (sometimes called a trend or drift), thus referred to as regionalized variables.^36^ A trend is a smooth but systematic non-random spatial variation.^37^ This spatial dependency, intertwined with variability, allows for the prediction of these variables, with associated uncertainty metrics, in unsampled locations, enhancing our understanding and modeling of natural phenomena. By acknowledging both the randomness and the structured trends in regionalized variables, we can better understand the dynamic of natural features, such as the case of the roughness of a rock fracture surfaces, critical for accurate fluid flow simulations.

A good example of regionalized variables is the case of how fluvio-lacustrine deposits reflect seasonal variations, with their thickness reflecting annual climate factors, such as average temperature, rainfall, and weather events. The spatial variables, therefore, combine randomness with structural trends (called a drift in the context of geostatistics). In the context of seasonal deposits, an example of structure phenomena is the “damped hole” effect in semi-variograms, where the drift is not linear but instead oscillates). By definition, random processes lack a deterministic mathematical description, but can nevertheless have a structure or correlation^37^.^38^ states that objects in closer proximity tend to share more comparable properties than those further apart, a principle derived from the intricate interplay of physical processes that shape the environment. Therefore, by collecting geological data at a specific point in time and space, we can model this environmental randomness and structure. This modeling, achieved through tools like the semi-variogram, enables us to predict regionalized variables at locations between our sampled data.

**Figure 1:**
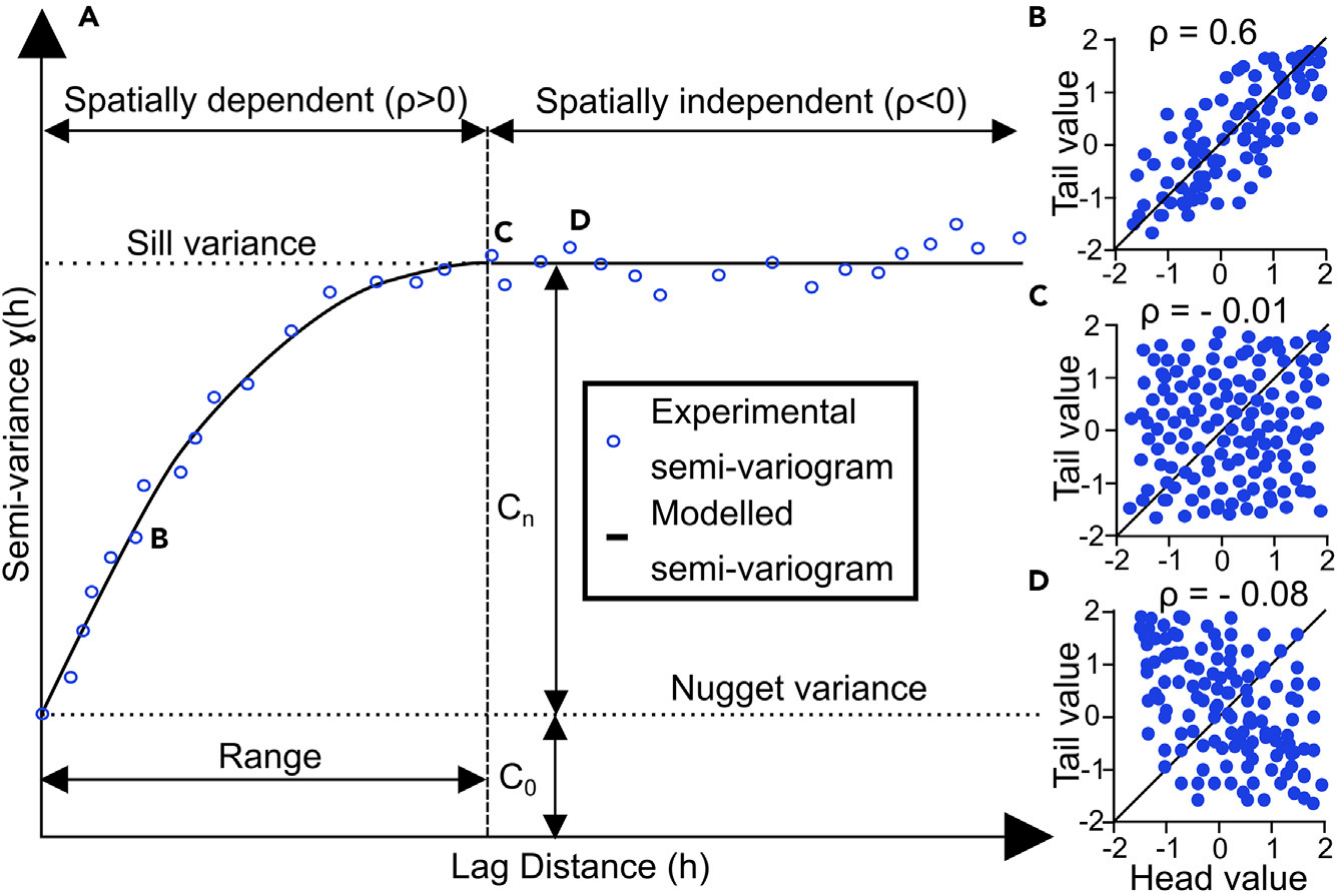
Anatomy of an experimental and modelled semi-variogram (A) Anatomy of a semi-variogram (adapted from^37^^,^^41^). An experimental semi-variogram is calculated through comparison of all point pairs in a particular direction for each lag (h) distance following Equation 1. Each point in the experimental variogram corresponds to the correlation coefficient (ρ) of the scatterplot along the x = y bisectrix between head and tail values of the vector between each point pair: for instance, points B, C, and D correspond to h-scatterplots (B), (C) and (D), respectively. The semi-variogram is a model of the semi-variance as a function of separation (lag, h) distance. The nugget corresponds to the semi-variance at the shortest measured separation distance. The sill variance corresponds to the maximum variance of the system. The sill allows the interpretation of the range. The range is the maximum separation distance until which there is correlation between data points (ρ < 0) and is interpreted as the separation distance at which the semi-variogram reaches the sill variance. C_0_ is the contribution of the nugget variance toward the total (sill) variance. C_n_ is the subsequent contributions of the n models necessary to model the experimental variogram. n is 1 if only one model is necessary to accurately model the system but can be 2 or more making it a nested model. See Figure 6 for an example of a nested model.

A semi-variogram is a fundamental tool in geostatistics, designed to measure the degree of spatial variability of a regionalized variable in a particular direction along increasing distances. Unlike the co-variance function - the complement of the semi-variance - which also describes spatial continuity, variograms are more commonly used due to their ability to model the average squared differences between data points as a function of their ‘lag’. The lag is the vector between the two points being compared which has a magnitude equal to the points’ separation distance and a particular direction. Variogram functions must be positive definite for their use in kriging and stochastic simulations because variances are intrinsically positive. This requirement that ensures the models are strictly positive, ergo physically plausible.^39^^,^^40^^,^^41^ To construct a variogram, we analyze the relationship between pairs of datapoints across a dataset in a particular direction, calculating the average *semi-variance* between all pairs at each separation distance (lag). The average *semi-variance* at each lag is in fact the complement of the correlation coefficient (ρ) of each lag (h-)scatterplot. This is a cross plot showing all pairs of data points separated by a particular lag in a particular direction, as demonstrated in Figure 1. The tail and head values refer to the lag vector connecting the pairs which are plotted in the X- and Y axis. ρ is the measurement of correlation of the point pairs to the X = Y bisectrix. If the tail and head values are the same, they will fall on the X = Y line and, if true for all point pairs, they would all fall along that line thus having a perfect positive correlation. When ρ is zero or negative it means the variance is maximum and there is no correlation between the points at those ranges. Figures 1C and 1D show ranges where no correlation exists, which happens above the sill. Notice the tail-head scatterplots analyzed exhibited linear correlation but this may not always be the case so a rank correlation coefficient might be better suited in other cases.^39^ This calculation is performed iteratively over a range of distances to build the experimental semi-variogram and, if necessary, directions, to reveal the spatial structure of the data. The resulting experimental semi-variogram graphically represents the semi-variance as a function of lag distance, helping to identify the primary directions of spatial continuity – i.e., the directions of maximum and minimum variance. The semi-variogram is therefore the graphical representation of half (hence “semi”^39^) the average squared difference between a property value pair for all measurement locations within a specific lag distance over all lag distances (Equation 1),

(Equation 1) $$\text{Semi}-\text{variance}\;\gamma \left(h\right)=\frac{1}{2N\left(h\right)}\;\sum_{i=1}^{N\left(h\right)}{\left(z_{x_{i}}-z_{x_{i}+h}\right)}^{2}$$

where γ is the semi-variance, h is the lag (separation distance), $z_{x_{i}}$ and $z_{x_{i}+h}$ are the values of the property at the tail and head of the vector, respectively. The semi-variogram is often modeled continuously, allowing for their use in geostatistical tools such as kriging. Many models are available such as the Gaussian and exponential models but only the spherical model (Equation 2) was used further due to better fits. An example of an experimental and a modeled semi-variograms along with its anatomy is shown in Figure 1.

(Equation 2) $$\text{Spherical}\;\text{Semi}-\text{variogram}\;\text{Model}\;\gamma \left(h\right)=\left\{ \begin{array}{c} 1.5h-0.5^{3}\;if\;h<1\\ 1\;if\;h\geq 1 \end{array}\right.$$

The *nugget effect* in variograms signifies an apparent discontinuity at very small lag distances, observed as initial variance that is greater than zero. This phenomenon can arise from several sources, including measurement error,^39^ the presence of micro-structures below the sampling resolution, or natural variability in the sampled variable.^41^ For example, in the context of mining, the *nugget effect* may be purely indicative of granulometric variations in gold and diamond deposits. But nugget effects might also reflect the existence of continuous micro-structures such as veins or high-grade infills.^42^ When applied to the study of rock fracture aperture, a nugget effect may be diagnostic of channeling below the scale of observation.

Understanding spatial continuity also necessitates assuming stationarity within the dataset, meaning the statistical properties of the variable, such as its mean, do not change over space or time. This assumption allows us to model the spatial correlation of a variable – like fracture aperture – based solely on distance and direction, a condition known as *intrinsic stationarity*.^36^ If a trend is present, thus violating the intrinsic hypothesis of stationarity, then *de-trending* is necessary.^43^^,^^44^ The process typically involves fitting (modeling) a trend surface through least squares and then analyzing the residuals – the difference between the observed values and the modeled trend – to ensure accurate variogram modeling.

Variograms are often directional, focusing on data pairs along specific orientations to uncover anisotropies or directional dependencies in the spatial structure of data. For example, in mining, variograms can elucidate the average shape and elongation directions of sediment micro-basins.^42^ If the dataset is isotropic, in other words having a uniform variance regardless of the direction it is measured, then a single variogram is sufficient to describe the spatial continuity of the data. However, real-world data often exhibit trends or directional dependencies (i.e., the data are anisotropic) becoming necessary to have multidimensional variograms (two for 2D data, three for 3D). Focusing on the 2D case, the first pass approach to finding these characteristic directions of anisotropy is to create a contour map of the sample variogram surface (variogram map).^39^ A more robust and more common approach to accurately finding these directions is to calculate the experimental semi-variograms for many directions. Interpreting their ranges will define the area up to which points have any correlation^39^: a spherical shape if the spatial continuity is isotropic and an ellipsoid otherwise (an ellipse of correlation in 2D).

### Kriging

Kriging is a class of algorithms used to predict a value of a property at an unsampled location making use of the spatial continuity of the values measured at the sampled locations. Kriging algorithms estimate the weights necessary to apply to the correlated sampled locations in order to predict the value at the unsampled location, while minimizing the error.^41^ In this work we opted for the ordinary kriging algorithm, often referred to as Best Linear Unbiased Estimator: Best because it finds the minimum squared error, linear because it uses linear square distances and unbiased because it forces the weights applied to the correlated measured sampled to equal 1. It is also applied to the situations where the trend or drift is not known, In opposition to the simple kriging.^40^

## Numerical model

Our study presents a numerical model that builds upon and refines the methodologies introduced by.^9^^,^^17^ While these foundational models employ elastoplastic coupled hydro-mechanical frameworks, our approach simplifies this complexity by adopting an elastic hydro-mechanical model. The choice to simplify the model in this way stems from the fact that a more competent lithology such as a gneiss is being used as well as the comparisons are performed between models under the same conditions, not against experimental data. Elastic behavior refers to models that recover their original properties once the physical inputs (stress, temperature, fluid pressure) are removed. Plastic behavior refers to models which do not recover to their initial state after the physical inputs are removed. This modification enables us to focus on the elastic behavior of the material, which is particularly relevant for understanding fluid flow through fractures under varying stress conditions. Additionally, it provides a simpler benchmark to establish the efficacy of the approach which can then be extrapolated to more complex models with more combinations of coupled processes. In fact,^9^ used fitting parameters for each of the elastic and plastic processes in their elasto-plastic model.

The model operates by first solving for the hydraulic behavior within the fracture. This is similar to the approach of,^9^^,^^17^ where the hydraulic solution is computed numerically to capture the dynamic fluid pressure distribution. Conversely, the mechanical response, specifically the deformation and stress distribution across the fracture surfaces, is derived through an analytical solution at the element level, thus allowing for precise calculation of stress-induced changes in aperture distribution.

**Figure 2:**
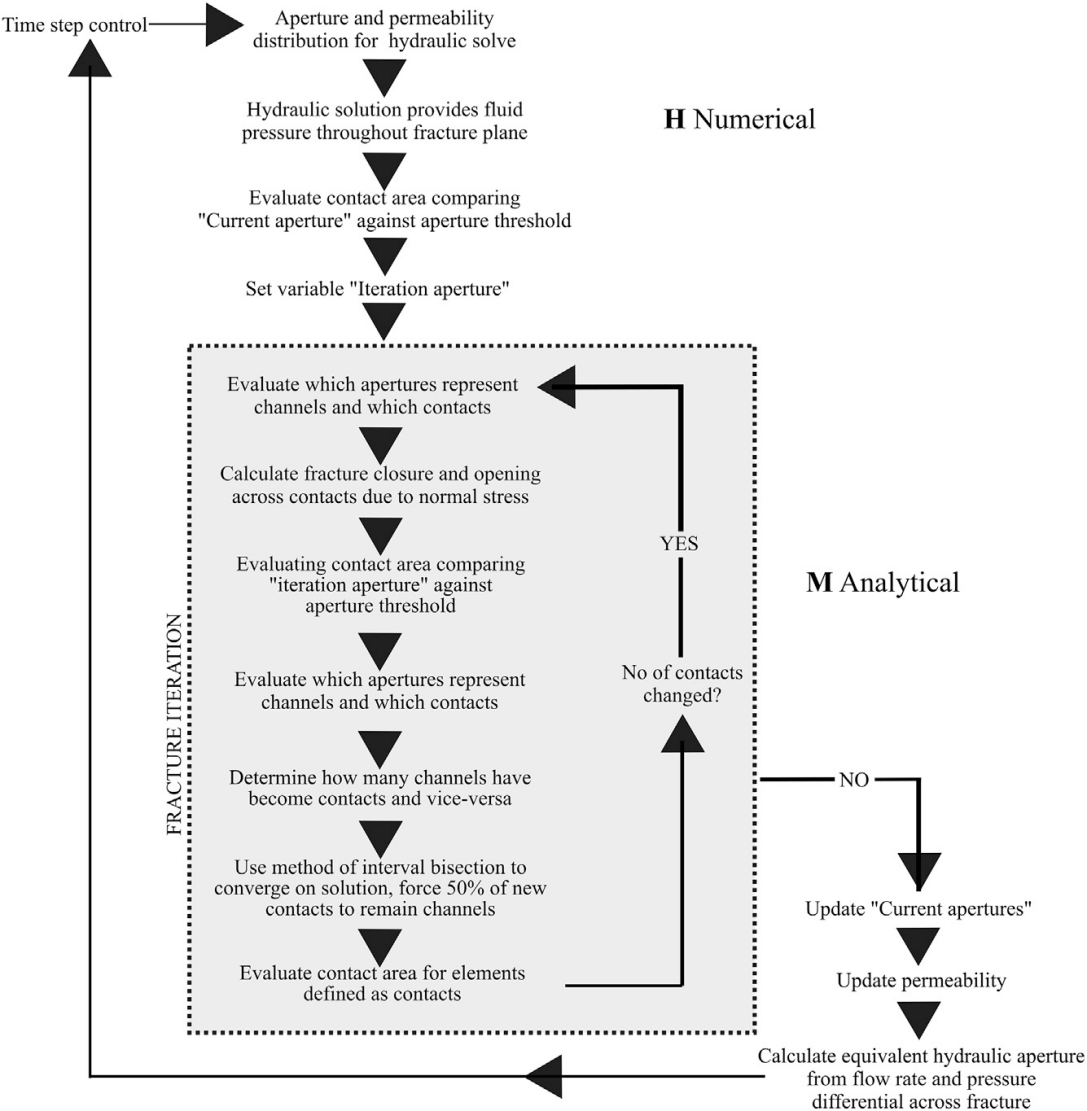
The conceptual model diagram of the fracture’s coupled hydro-mechanical numerical model

A schematic representation of the model workflow is represented in Figure 2. The process begins with the input of aperture distribution data, either from previous iterations or the initial condition at the commencement of the simulation. This data informs the calculation of the permeability field across the fracture, laying the groundwork for simulating fluid flow. Subsequently, the fluid pressure field is determined numerically, leveraging the permeability field to understand the hydrodynamics within the fracture. A critical aspect of our model is the evaluation of the contact area within the facture surface. This is achieved by applying a user-defined aperture threshold, normally chosen as the minimum aperture observed in the distribution, which aids in identifying regions of potential fluid passage or blockage and serves as the control variable for moving onto the next iteration. The outcome of this evaluation is stored as the iteration aperture field, providing the baseline for subsequent model iterations.

In each timestep, we simulate the application of normal stress across the fracture plane. This stress alters the fracture aperture distribution, a process calculated analytically at the level of individual mesh elements. Through this technique, we assess the evolution of fluid channels and contact points, considering the effects of stress on opening or closing apertures. By comparing the contact area derived from the current solution against that at the start of the next iteration – factoring in the defined aperture threshold – we can gauge the progression of fracture elastic deformation. The model iterates through this process, adjusting for the number of contacts and aperture distribution until a stable state is achieved, indicated by the consistency of contact numbers between two consecutive iterations. This stability criterion prompts the model to update the apertures and permeability fields, marking the transition to the next timestep.

**Figure 3:**
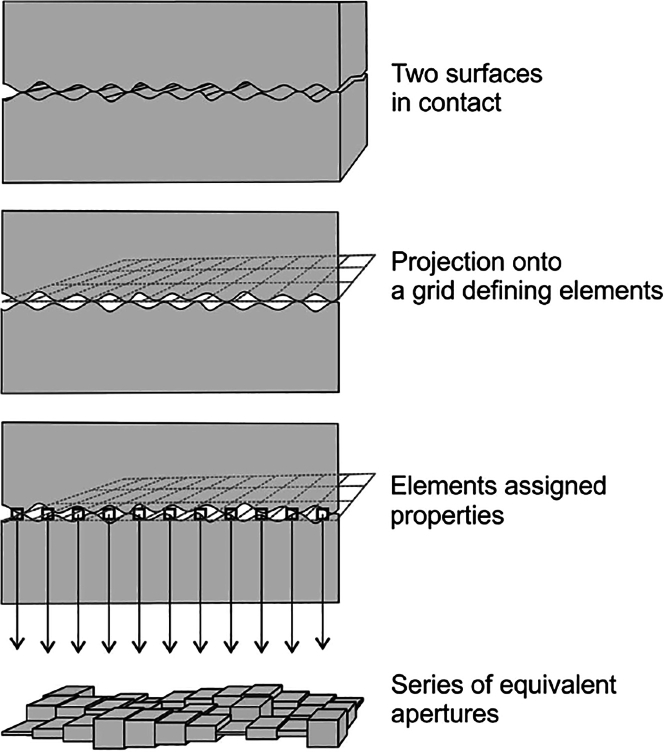
Aperture distribution mapped onto a discretised mesh on an idealised fracture plane^9^

Our adaptation of the conceptual models into a practical numerical framework involves projecting the apertures between the two fracture surfaces onto a discretized, idealized fracture plane as can be seen in Figure 3. This projection serves as the basis for simulating fluid flow and mechanical interaction within the fracture. Similarly to the adaptation of,^17^ we include two fringes of high permeability on the model’s flow input and output to distribute the fluid pressure evenly thus avoiding pressure artefact. Unique to our approach is the dual resolution employed – fine and coarse – in parallel models to assess the impact of resolution on simulation outcomes. Additionally, we compare the effectiveness of two aperture distribution upscaling methods as described in the previous section.

The permeability for each mesh element is calculated using the cubic law, applied locally for laminar flow conditions. According to Equation 3 derived from,^9^^,^^17^^,^^20^ the permeability $K_{i,j}^{elem}$ for the element located at grid coordinates i,j is calculated using the element aperture, e,as follows:

(Equation 3) $$\text{Cubic}\;\text{law}\;K_{i,j}^{elem}=\frac{e^{2}}{12}$$

The minimum permeability in each model is dependent on the geometry of the mesh and the upscaling method employed. The minimum permeability in the fine models was set to zero. This signifies that element in contact, i.e., below or equal the minimum aperture in the distribution, do not allow flow. The minimum permeability in the coarse models is set to 1e^−15^m^2^: This is the permeability threshold from which the coarse average model starts having high enough fluid pressures at the element level to surpass the confining stress. This causes the OpenGeoSys (OGS)^45^ code to set the fluid pressure to zero as it is not optimized to cope with such conditions. This coping mechanism of the code would incur in artifacts in the results which would make the comparisons more difficult. The coarse kriging model coped with lower minimum permeabilities (in the order of 1e^−17^m^2^) but for the sake of comparison, the minimum permeability value of the average coarse model was kept as the coarse averaging method. This influences fluid pressure results in that, even with apparent closure, numerically the model allows for fluid to pass through to the outlet, ensuring numerical stability in some cases, particularly when the fracture channels close toward the outlet.

The models presented here are conceptualized from experiments conducted at the GREAT cell.^11^ The GREAT cell is an apparatus capable of reproducing subsurface stress conditions equivalent of up to 3.5km and fluid flow on 200mm long, 194mm diameter cylindrical rock samples with fracture networks. It exerts axial pressure on the top and base of the sample through a hydraulic press (Figure S1). The radial stresses are applied through eight opposing pairs of hydraulic cushions which can be independently set (Figure S2) to create true triaxial stress conditions within the sample. Model conditions, consistent across all simulations, are adapted from experiments conducted in the GREAT Cell from work in preparation for publication. The experiment is conceptualized as a planar fracture in which the y axis is aligned with the direction of $\sigma_{1}$ and $\sigma_{2}$ also parallel to the fracture plane (along the x axis), meaning $\sigma_{3}$ starts normal to the fracture. The application of normal stress across the fracture plane is informed by stress rotations experiments, featuring the second and third principal stresses ($\sigma_{2}$ and $\sigma_{3}$ ) rotating around the idealised fracture plane, anchored by a constant first principal stress ($\sigma_{1}$) of 12MPa, parallel and static to the fracture orientation throughout the whole model.

The stress rotation scenario unfolds over several stages, each involving a 22.5° rotation of the $\sigma_{2}/\sigma_{3}$ stresses around the $\sigma_{1}$ axis. This rotation continues until a full 180° turn is completed, with $\sigma_{2}$ returning parallel to the fracture plane. Within each stage, we implement seven sub-stages where the magnitude of $\sigma_{2}$ is incrementally increased from 6MPa ($\sigma_{3}$ value) to 12MPa ($\sigma_{1}$ value), in 1MPa steps. This detailed stress modulation, summarized in Table 1, uses directional cosines from accurate normal stress calculation on the fracture plane, as outlined by.^46^^,^^47^ Further information on the experimental procedure and results can be found in.^10^

**Table 1:** Normal stress applied to the idealized fracture plane at each moment in time

Time (s) of application of stress	σ_n_ (MPa)	Time (s) of application of stress	σ_n_ (MPa)	Time (s) of application of stress	σ_n_ (MPa)	Time (s) of application of stress	σ_n_ (MPa)
**0**	6.00	**4800**	7.00	**9600**	10.00	**14400**	9.00
**300**	6.00	**5100**	7.50	**9900**	11.00	**14700**	6.00
**600**	6.00	**5400**	8.00	**10200**	12.00	**15000**	6.15
**900**	6.00	**5700**	8.50	**10500**	6.00	**15300**	6.29
**1200**	6.00	**6000**	9.00	**10800**	6.85	**15600**	6.44
**1500**	6.00	**6300**	6.00	**11100**	7.71	**15900**	6.59
**1800**	6.00	**6600**	6.85	**11400**	8.56	**16200**	6.73
**2100**	6.00	**6900**	7.71	**11700**	9.41	**16500**	6.88
**2400**	6.15	**7200**	8.65	**12000**	10.30	**16800**	6.00
**2700**	6.29	**7500**	9.41	**12300**	11.10	**17100**	6.00
**3000**	6.44	**7800**	10.30	**12600**	6.00	**17400**	6.00
**3300**	6.59	**8100**	11.10	**12900**	6.50	**17700**	6.00
**3600**	6.73	**8400**	6.00	**13200**	7.00	**18000**	6.00
**3900**	6.88	**8700**	7.00	**13500**	7.50	**18300**	6.00
**4200**	6.00	**9000**	8.00	**13800**	8.00	–	–
**4500**	6.50	**9300**	9.00	**14100**	8.50	–	–

Furthermore, the hydraulic conditions feature a constant backpressure of 4.89MPa (Dirichlet boundary condition) at the model outlet and introducing a fluid flux source term of 5 mL/m at the inlet. These are applied to two fringes of high permeability added on either side of the numerical model’s mesh to prevent high pressure artifacts.

## Results and discussion

### Spatial continuity analysis

**Figure 4:**
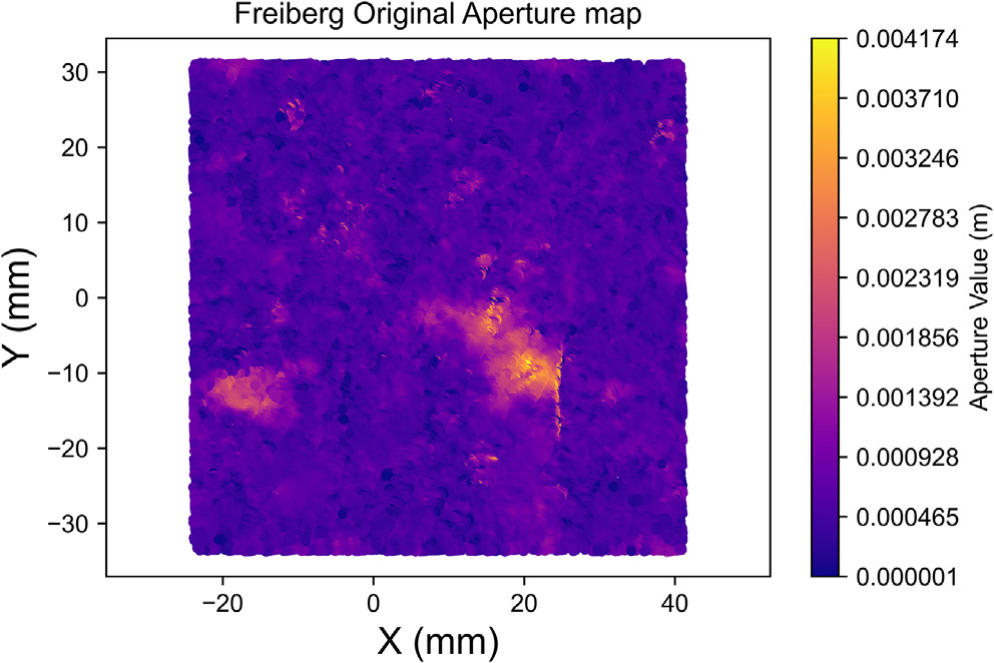
Closer caption of the Freiberg gneiss middle-section aperture field

**Figure 5:**
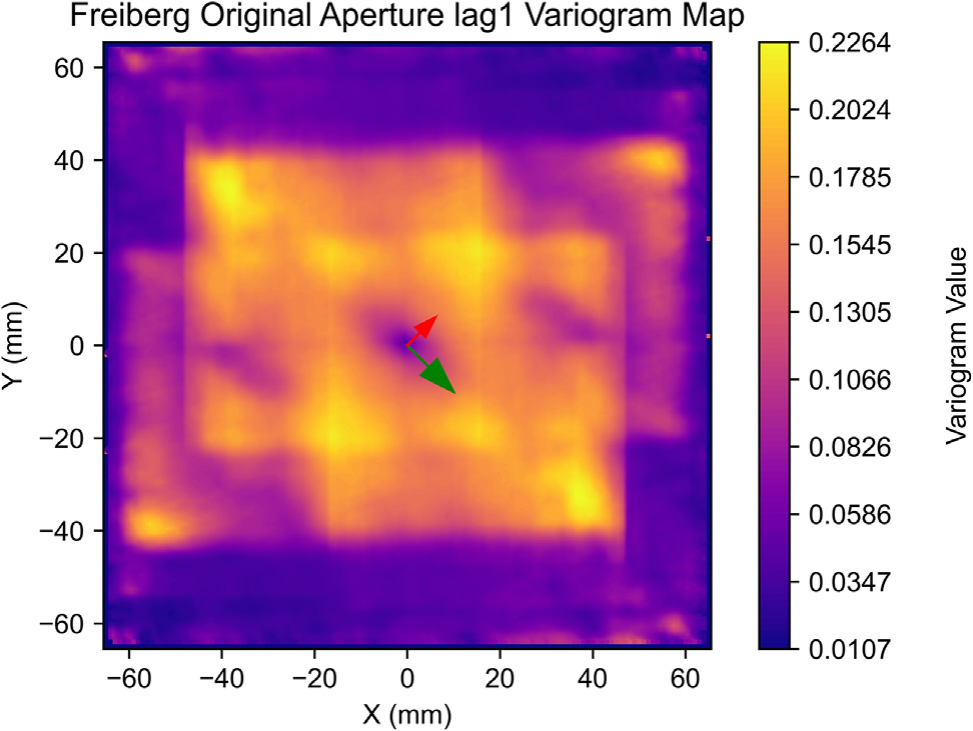
Variogram map of the Freiberg gneiss middle-section aperture field Green and red arrows depict the preliminary interpreted major and minor continuity directions and magnitudes, respectively.

**Figure 6:**
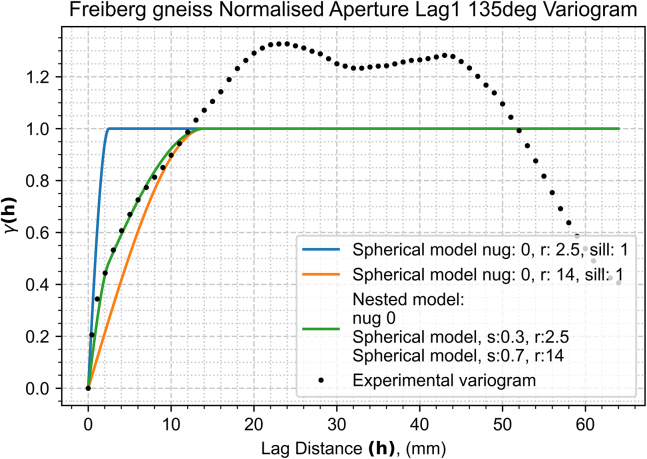
Major continuity direction experimental and modeled semi-variograms for the Freiberg gneiss middle-section aperture field corresponding to 135° azimuth The cyclicity observed beyond the sill is possibly indicative of channeling phenomena within the aperture field. The colored lines represent continuous semi-variogram models The blue model better represents the shorter lags’ semi-variance but underestimates it at longer lags. The orange model overestimates the shorter lags’ semi-variance but does a better job at modeling the longer separation distances. The green model is a nested spherical model which represents the experimental semi-variogram much more accurately than the other two simple models shown in both short and long lags.

**Figure 7:**
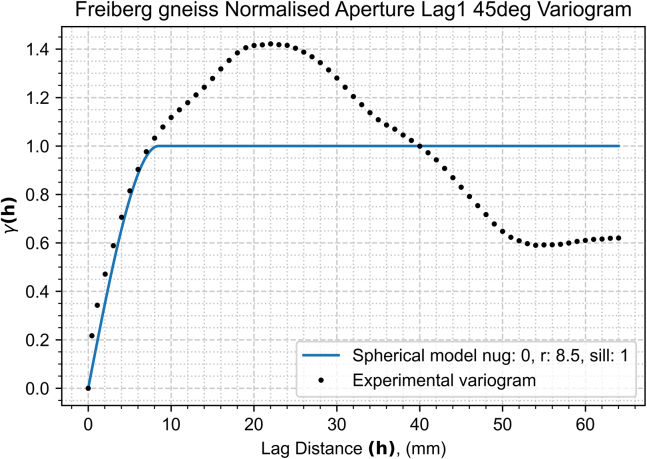
Minor continuity direction experimental and modeled semi-variograms for the Freiberg gneiss middle-section aperture field corresponding to 45° azimuth The cyclicity observed beyond the interpreted range is possibly indicative of channeling phenomena within the aperture field. The colored line represents a continuous semi-variogram model.

In our detailed exploration of spatial continuity with a newly compiled dataset (Spatial continuity theory), we embark on a comprehensive variogram analysis. This analysis is pivotal in unveiling the underlying spatial characteristics of the fracture aperture field. The aperture field (Figure 4) underwent spatial continuity analysis to generate an aperture variogram map (Figure 5) and experimental semi-variograms for each characteristic direction (Figures 6 and 7), set 22.5° apart. The analysis parameters used – 1 mm lag, lag tolerance at half-size for partial overlap, and azimuth tolerance of 22.5° – were selected to optimize accuracy and computational efficiency, yielding 65 lags in each direction based on the data dimensions, informed by best practices.^37^^,^^39^^,^^41^^,^^48^ With the variogram map (Figure 5) serving as a foundational element in our process and employing the parameters previously outlined, we identify major and minor continuity directions at approximately 135° and 45°, respectively.

These orientations, crucial for understanding the fracture heterogeneity, are robustly supported by the experimental and modeled semi-variograms displayed in Figures 6 and 7.

Acknowledging the computational intensity of our approach, we strive to calculate experimental semi-variograms across a spectrum of directions, separated by increments of 22.5°, as allowed by the computational resources available for this work. This exhaustive approach, while comprehensive, necessitates a focus on the two principal directions for clarity in the presentation and analysis. Notably, the normalization process applied to our data defined the sill variance to be a standard unit (unit sill), a methodological choice aligned with established practices in the field by.^41^^,^^48^

In our analysis, as demonstrated in the semi-variograms of both major and minor continuity directions (Figures 6 and 7), a stable sill is observed, confirming second-order stationarity, and negating the need for de-trending. This finding validates the suitability of our dataset for spatial continuity analysis without further modification for trends.

Our findings reveal a close alignment of the experimental semi-variograms with the spherical model (Equation 2), signifying its effectiveness in encapsulating the spatial continuity of our dataset. This model outperformed other models, such as exponential, Gaussian, and power models. A particularly insightful comparison is presented in Figure 6, where a nested spherical model (illustrated in green) demonstrates enhanced fidelity in representing the major continuity direction, outperforming simpler models (blue and orange) that struggle with accurately modeling semi-variance across the range of lags. The choice of parameters (azimuth, nugget, range and sill) for the kriging algorithm are provided by the best fitted models from Figures 6 and 7.

Despite the nested model’s superior representation accuracy, its complexity restricted its application within our kriging algorithm, detailed in Section 2.1. This limitation underscores a critical modeling principle advocated by,^49^ the value of simplicity. Overfitting, a potential pitfall of overly complex models, often detracts from rather than enhances predictive accuracy. Our analysis, therefore, adheres to a simpler modeling approach, balancing precision with practical applicability.

An interesting aspect of our analysis is the observation of a wavy variance pattern in the modeled semi-variograms (Figures 6 and 7), extending beyond the established range with increasing lag distances. This pattern suggests the presence of cyclicity within the data, possibly indicative of channeling phenomena within the aperture field. Such cyclicity, reflecting periodic or repeating structures, could have significant implications for understanding fluid dynamics and mechanical stability within fractured media. Another point of notice is that in both cases the slope of the variance of the apertures for short lags in the sub-millimeter scale is remarkably high. In other words, aperture values in the immediate vicinity of each other tend to be quite different. This emphasizes the fact that sub-millimeter scale may have a substantial impact on the behavior of flow in fractures.

This phase of our investigation not only reinforces the foundational understanding of spatial continuity in geological fractures but also opens avenues for further research. The observed cyclicity, in particular, merits deeper exploration to elucidate its origins and impacts on fracture behavior under varying stress conditions. Our work thus lays a groundwork for future studies, aiming to refine the predictive capabilities of numerical models concerning fractured rock hydrodynamics.

### Finite element method (FEM) numerical model

In this work, we used an FEM numerical model to examine the coupled hydro-mechanical processes occurring within a fracture, using both coarse and fine mesh resolution alongside two distinct upscaling methods: averaging and kriging.

**Figure 8:**
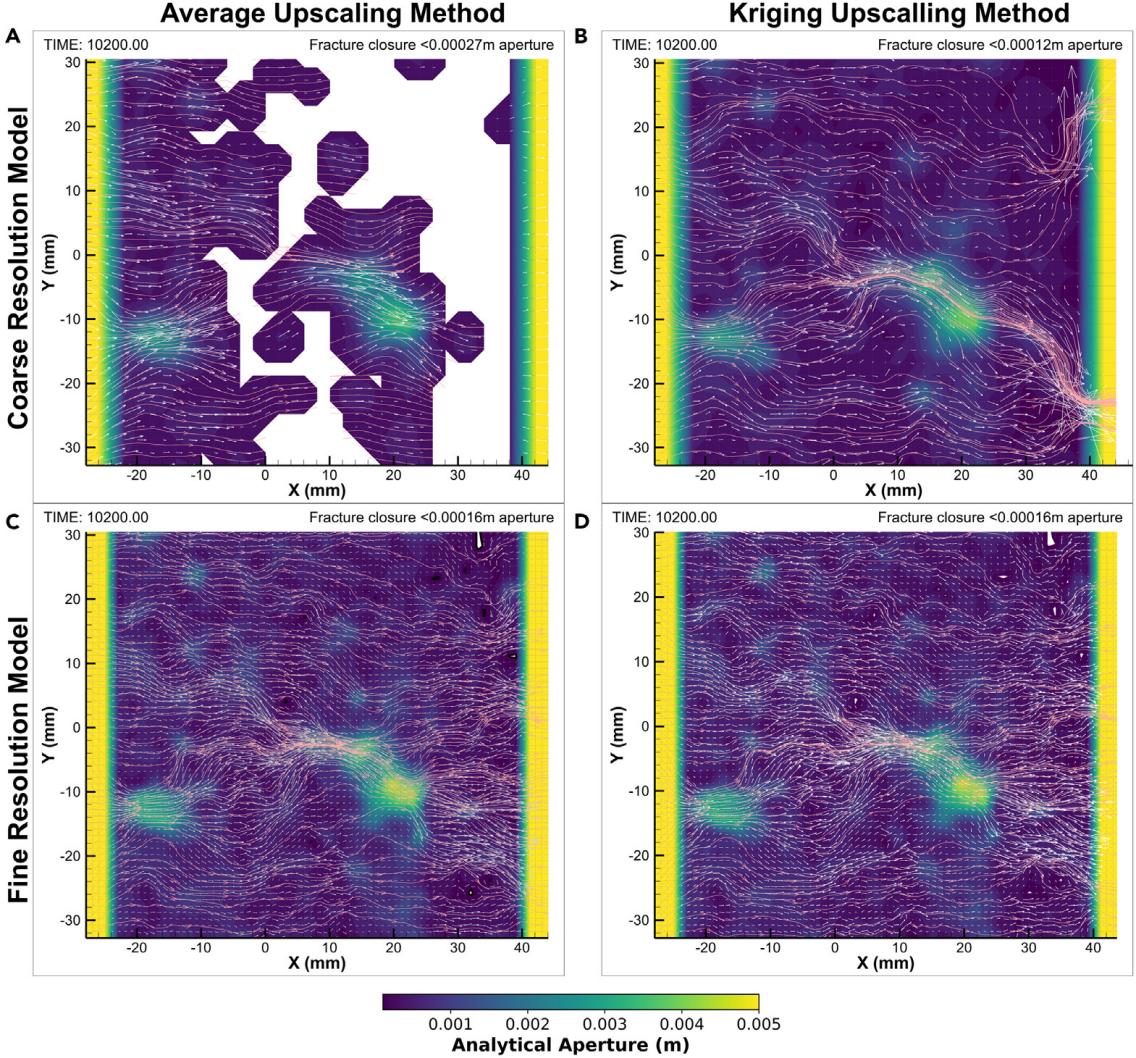
Model comparison The top row shows the coarse model (32x32 elements) and the bottom row the fine model (64x64 elements) – The 2 and 4 fringes’ elements in either side (coarse and fine models respectively) are not accounted for. Left and right columns display the averaging method (arithmetic averaging and kriging, respectively). The color map represents the hydraulic aperture, the white vectors the fluid velocity and the pink arrows the streamlines. The models are blanked where the fracture is deemed in contact across the two faces. White vectors represent the fluid flow velocity. The coarse models have different vector magnitude scales whereas the fine models are in scale.

The choice of upscaling method and mesh geometry significantly influenced the aperture field values and spatial distribution, leading to variations in the minimum aperture thresholds across models. The coarse models have elements of roughly 2 mm^2^ whereas the fine models' elements are approximately 1 mm^2^. Given the spatial continuity ranges of 14 mm and 8.5 mm in the major (135°) and minor (45°) continuity directions respectively, the ellipse of correlation extends far beyond the element is predicting. Using our modeled semi-variograms, in this particular case 80% of the correlation in either direction is encapsulated in the 8 mm closest to the center of the element, nearly 4 times the size of the current coarse model element size. However, for a more robust understanding of the optimal balance between the spatial continuity ranges and directions and the size and geometry of the mesh requires further investigation. Interestingly, the fine mesh models, due to their higher resolution, exhibited similar performance across both upscaling methods, necessitating a unified aperture threshold, therefore demonstrating consistency between the methods, and building confidence in the original upscaling method discussed in this paper. Conversely, in the coarse mesh models, the differences between the upscaling methods became pronounced, with the kriging method yielding an aperture threshold more akin to that of the fine models compared to the averaging method. The values of the utilized aperture closure thresholds, defined in Figure 8, are 0.16 mm for the fine models, 0.12 mm for the coarse kriging and 0.27 mm for the coarse averaging methods.

It also emphasizes the possible implications of using less optimal standard upscaling methods such as averaging. The transition point at which one upscaling method surpasses the other in terms of effectiveness is not straightforward and likely depends on the inherent spatial continuity of the dataset. Further research is required to elucidate this relationship more clearly. Despite the inherent loss of sub-grid scale information in discretized fracture models, our goal with the kriging approach is 2-fold: to integrate spatial continuity into our model and to encapsulate sub-grid scale information to the fullest extent permitted by the dataset.

**Figure 9:**
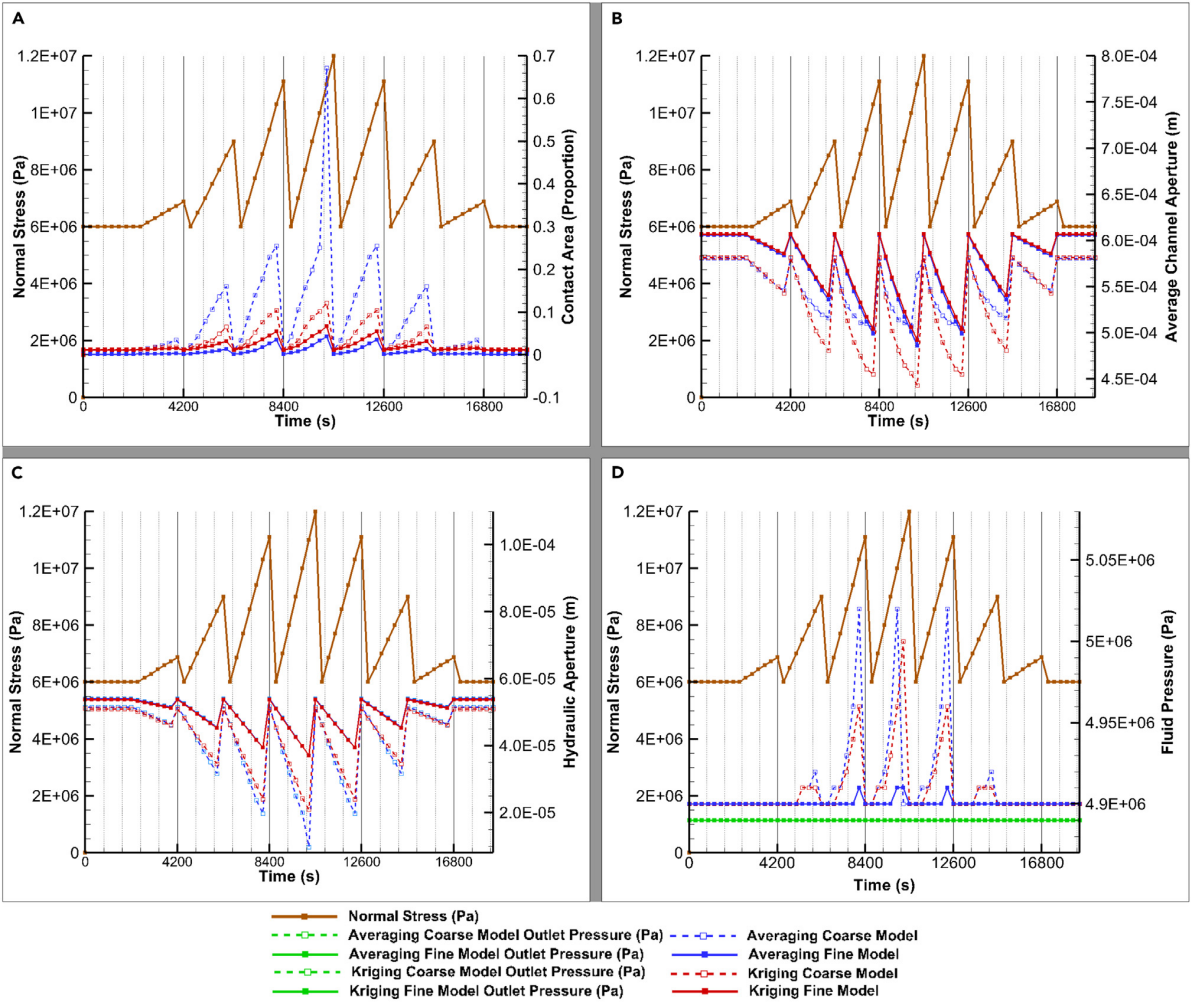
Modeling results Full lines with filled symbols represent the fine models whereas the dashed lines with unfilled symbols represent the coarse models. Red lines represent kriging and blue lines averaging upscaling methods. (A) Contact Area (%). (B) Average Channel Aperture (m). (C) Hydraulic aperture (m). (D) Fluid inlet and outlet (green lines) pressures (Pa). Fluid outlet pressures share the same color for all models since they are all affected by boundary conditions and all overlap, shown here only for demonstrating numerical stability.

Comparisons between fine mesh models (solid lines with filled symbols in Figure 9) affirm their alignment across all evaluated metrics. Sub-figures in Figure 9 further detail these comparisons, highlighting variations in fluid inlet pressure, hydraulic aperture, average channel aperture, and contact area among the models. While comparing the upscaling methods through the coarse mesh models (dashed lines and unfilled symbols), the kriging upscaling method (red lines) consistently approximates the benchmark more than the average method (blue lines) across these metrics. Albeit with some deviations in specific measures such as the average channel aperture. These distinctions aid in visual comparison and underscores the mesh resolution impact on model outcomes. It is noteworthy that the outlet pressures remain consistent across the models, attributed to the imposition of a constant boundary condition. This uniformity ensures a controlled comparison of the models' performance under identical hydraulic boundary conditions.

Focusing on Figure 9A, we examine the contact area, an essential metric for understanding fracture behavior. Here, the coarse mesh model using kriging outperforms the averaging approach, offering a more accurate approximation of the fine mesh models’ contact areas. While the coarse averaging model significantly overestimates contact area—to an order of magnitude—the kriged model, though still overestimating, remains much closer to the fine mesh predictions. This consistency in behavior between models, despite the overestimation, further illustrates the influence of mesh resolution and upscaling method on capturing key aspects of fracture mechanics. Additionally,**^9^** suggested pressure solution, a mechanical-chemical process influencing the dissolution of mineral species driven by stress, as the key mechanism for aperture reduction. Pressure solution mainly occurs in asperities in contact where the stress is concentrated. Hence, an upscaling method that is more accurate in calculating the initial contact area will hopefully render more accurate THMC coupled processes models.

In Figure 9B shifts focus to the average channel (non-closed element) aperture, where the coarse mesh model employing kriging closely mirrors the response patterns of the fine mesh models across various stress conditions. On the other hand, the coarse mesh model with averaging diverges from the fine mesh patterns mostly during the three primary closure stages, although it aligns more closely under lower normal stress conditions. Even when the behavior diverges, the absolute values are more similar to the benchmarks than the kriging coarse model. This apparent overperformance of the average coarse model can be explained by the larger aperture distribution when compared to the kriging coarse model aperture distribution, a consequence of the averaging upscaling method. Figure S3 illustrates this point by showing the average coarse model having a distribution more focused around the mean (lower standard deviation) whereas the kriging coarse model distribution has a wider distribution around the mean (higher standard deviation). This means the lower standard deviation of the averaging coarse model causes larger contact area. Simultaneously, the generally larger apertures cause the larger average channel aperture. This nuanced difference highlights the impact of upscaling method on model performance under varying mechanical stresses.

Figure 9C, we illustrate the hydraulic aperture across the models, continuing the same visual conventions established in Figure 9A for distinguishing between coarse/fine and averaged/kriged models. Notably, the fine mesh models, regardless of the upscaling method – average or kriging – exhibit a strong concordance in hydraulic aperture values, underscoring their reliability as a benchmark. Conversely, the hydraulic aperture derived from the coarse mesh model using kriging deviates less significantly from this benchmark compared to its averaging counterpart, albeit marginally. Despite this divergence, both coarse models effectively capture the overarching trends observed in the fine mesh models, demonstrating their capacity to reflect the primary hydraulic behaviors.

Turning to inlet pressures in Figure 9D, which are influenced by the compression of the fracture, we observe a subtle yet significant distinction in the model behavior. The coarse mesh model using averaging upscaling tends to overestimate inlet pressures compared to its kriging counterpart, albeit by a minimal difference of only a few kilopascals (KPa). This discrepancy underscores the sensitivity of the models to the upscaling method employed, particularly in scenarios of fracture compression where spatial resolution and upscaling technique subtly influence the pressure estimations. The outlet pressures from all models overlap, reinforcing the boundary conditions is being applied correctly and corroborating the robustness of the models.

As depicted in Figure 8, the models can be visualized with coarse and fine mesh resolution on the top and bottom rows, respectively, and with averaging and kriging methods on the left and right columns. Comparative statistics between the raw apertures and initial coarse averaged and kriged models are offered: for the kriged model, Figure S4 shows the error map between the two and Figure S5 shows a cross-plot with root-mean-square error (RMSE) and coefficient of determination (r^2^). Figure S6 and SM7 show the respective information relating to the averaging method coarse model. In all cases, the kriged model show closer values to the initial high-resolution aperture data.

Blanked sections within each model represent areas where the aperture meets or falls below the model-specific threshold, indicating contact between the two fracture surfaces. The time of 10200 s, chosen for illustration, marks the peak of normal stress exerted on the fracture, aligning $\sigma_{1}$ (12 MPa) parallel to the y axis and $\sigma_{3}$ (6 MPa) parallel to the x axis, with $\sigma_{2}$ (12 MPa) acting perpendicular to the fracture plane. The kriging method’s advantages are evidenced by closer resemblances in aperture and streamlines, to those observed in the finer mesh models, despite the expected reduction in resolution. Notably, the coarse mesh model using kriging did not exhibit as much fracture closure (blanked portions) as the coarse average model, exhibiting a fracture closure closer to the fine mesh models hence underscoring a more robust solution compared to the averaging method, which failed under the same conditions at the model outlines.

When compared to the benchmark fine models, the excessive fracture closure at the outlet of the coarse model using the average upscaling method unveils a weakness in the standard upscaling methods at particular upscaling scales. Elements in contact force the fluid volume to flow to neighboring elements which counteracts the normal stress being applied. This creates a lateral (perpendicular to the flow direction) aperture increase effect, which smooths out the channels. This is observed in the coarse average model. Due to the similar contact area in all other models, the channeling is similar in all cases. In the coarse average case, this excessive closure causes the complete flow cessation toward the outlet, which illustrates the care needed when using such methods. The distribution’s lower spread (similarity of values) across the fracture (Figure S3), may explain this behavior: it forces elements to contact at similar confining pressures hence closing the fracture along the outlet since the fluid pressures tend to be more similar perpendicular to the fluid flow direction. Additionally, the streamlines of this model display a straighter behavior, indicative of a smoother aperture field and possibly a consequence of the increased fluid pressure as explained above. Finally, the standard method model shows streamline convergence toward one single point, an indication of a sharp difference on either side of the flow channel. The convergence of the streamlines is indicative of an increase in flow velocity in the channel. This, in turn, may have large implications on dissolution rates in chemical coupled processes models since more rapid flux leads to decreasing amounts of dissolution with time by reducing the contact time of the fluid with the fracture faces.^9^ Once again, these observations may be explained by the lower standard deviation of the distribution forcing the fluid to converge into the relatively higher aperture conglomerate of elements thus forming a lower number of channels. The standard deviation of the aperture distribution may therefore be a good measure of the “discretization” level of a fracture.

On the other hand, the levels of detail in the aperture field and the streamlines are kept at the kriged coarse model when compared to the fine models, demonstrating a superior upscaling method even with the same upscaling level. Here, the streamlines keep their variable behavior, suggesting a sub-grid scale component of the aperture field. This sub-grid scale at the level of the coarse model can be observed in the fine models. Finally, the convergence of the streamlines along the main channel in the kriged coarse model demonstrates a more gradual stepwise nature, indicative of the incorporation of the sub-grid scale into the aperture field despite the similar upscaling resolution as with the coarse average model.

In the past few years,^34^^,^^35^ applied the spatial continuity concept for modeling fracture permeability fields. Just recently,^50^ introduces permeability estimation for rough fractures using persistent homology however the directions seem arbitrarily chosen depending on the sample collection. This work provides an advancement to understanding the implications of including aperture field’s spatial continuity information on coupled THMC processes numerical models results. Its novel approach allows for incorporating sub-scale information into coarser numerical models therefore helping reduce modeling complexity and consequently computation times.

### Limitations of the study

The method is computationally and memory expensive, requiring an N! number of operations when using the maximum resolution – where N is the number of points. However, measures can be taken to alleviate computation such as using a larger starting separation distance. Additionally, it requires a certain degree of spatial continuity knowledge and experience. It is also subject to the informed choices on the experimental semi-variograms analysis parameters and spatial continuity parameters used to model them.

### Conclusions

The generally used statistical representations of fracture rough surfaces, aperture, or permeability distributions often overlook the true statistical directionality of the data which may result in a poor representation of the roughness distribution or aperture field in 2D and thus in poor models.

This methodology analyses the directionality of the dataset in all dimensions, as opposed to simple 2D profiles, arbitrarily chosen or complete negligence of directionality by other methods, through spatial continuity analysis methods such as the semi-variogram.

This work not only characterizes the fracture portion aperture’s directional variability using semi-variograms but unveils its spatial correlation that other methods cannot.

This study therefore presents a new methodology to characterize fracture aperture fields (or surface roughness if necessary) random functions deterministically using only two modeled functions; whereas other methods such as the Fourier transform require at least 3 parameters (wavelength, amplitude, and phase)^25^ multiplied by as many frequencies the investigator requires to achieve a reasonable approximation. This characterization includes correlation between data points and directionality in that correlation. Additionally, it explicitly encompasses information regarding all scales of measurement.

In doing so, this methodology demonstrates applicability to numerical simulations methods, such as in coupled THMC processes, by including sub-grid scale information in such models. As demonstrated in this work, rendering objectively better results than methods that neglect spatial continuity of the data, thus lowering models’ complexity as well as computation time and power in running those models. Additionally, numerical models upscaled using traditional (averaging) methods show fracture closure artifacts and aperture fields deviations from the validatory models. Differently our models, incorporating spatial continuity, do not show such artifacts.

To the best of our knowledge, this is the first study to apply semi-variograms to a rough fracture aperture field in order to characterize the true spatial continuity directions, not only in interpreted or arbitrarily chosen directions. It is also the first study to include spatial continuity information in numerical models as an upscaling method and assessing its implications by comparing the results with models upscaled using standard upscaling methods.

By characterizing the spatial continuity of the fracture data, the correlation between the asperities or apertures is defined. Within its limitations, this method can be useful to incorporate the sub-grid scale information into the model without having to lower the mesh resolution, therefore rendering better results than simple mesh upscaling methods. Possible applications of this technique are (1) to know *a priori* what the average direction of flow will be; (2) preferential directions of shear or directions of dilation vs. shear displacement ratios; and/or (3) extrapolation of the fracture surface or aperture distribution away from collected datapoints. However, more research is required to understand the possible applications and their limitations.

Future work should be focused on (1) creating HM numerical models to analyze relationships between the flow directions and permeabilities with the variogram analyses; (2) creating HM numerical models to investigate the relationship between the peak shear stress and the directions of major and minor continuities; (3) investigating the relationship between fractures dilations (aperture fields development) as function of shear displacements in the directions of major and minor continuities of the roughness; (4) applying fracture surfaces’ variogram analyses to blind predict outside where data are available; (5) creating a database of spatial continuity analysis on a variety of fracture types (natural/artificial, tension/compression/shear) and lithologies. Specifically related to the upscaling method, a way forward is to analyze the relationship between mesh size, element geometry and orientation with regards to the spatial continuity in order to maximize the overlap between the correlation ellipse and the elements areas. This will provide a better understanding the optimal assemblage in order to maximize the spatial continuity statistics in predicting the most accurate values for each element.

## Resource availability

### Lead contact

Further information and requests for resources should be directed to and will be fulfilled by the lead contact, G.C. (g.cunha@ed.ac.uk, goncalo.bc9@gmail.com).

### Materials availability

This study did not generate any new materials.

### Data and code availability

• All data (fracture data and models) reported in this paper are available in an online publicly accessible GitHub repository (refer to the key resources table).
• All code necessary to reproduce the results is detailed in the key resources table.
• Any additional information required to reanalyze the data reported in this paper is available from the lead contact upon request.

## Acknowledgments

This research was funded by a combination of the The University of Edinburgh, Quintessa Ltd, the Nuclear Waste Services UK and by the EPSRC Smart Pulses for Subsurface Engineering (EP/S005560/1). See details at.

https://gow.epsrc.ukri.org/NGBOViewGrant.aspx?GrantRef=EP/S005560/1.

The research leading to these results was conducted within the context of the international DECOVALEX Project (http://www.decovalex.org).

## Author contributions

**Conceptualization**, G.B.C., C.I.M., A.B., and A.F.H.; **Methodology**, G.B.C., C.I.M., A.B., and A.F.H.; **Software**, G.B.C. and R.E.R.; **Validation**, G.B.C., C.I.M., A.F.H., and R.E.R; **Investigation**, G.B.C.; **Writing – Original Draft**, G.B.C.; **Writing – Review & Editing**, G.B.C., R.E.R., A.F.H., A.B., and C.I.M.; **Funding Acquisition**, C.I.M. and A.B.; **Resources**, C.I.M. and A.F.H.; **Data Curation**, G.B.C.; **Supervision**, C.I.M., A.B., A.F.H., and R.E.R.

## Declaration of interests

The authors declare no competing interests.

## STAR★Methods

### Key resources table

**Table:** 

REAGENT or RESOURCE	SOURCE	IDENTIFIER
**Deposited data**		

Fracture data and models	This paper.	https://github.com/g9cunha/Advancements-in-coupled-processes-numerical-models-upscaling-using-spatial-continuity/tree/main

**Software and algorithms**		

Code to calculate aperture (difference) between two fracture rough surfaces	This paper. Cunha ^51^^,^^52^	https://doi.org/10.5281/zenodo.10707260
Code to upscale fracture data into the GINA format (.msh file) numerical model mesh	This paper. Cunha ^51^^,^^52^	https://doi.org/10.5281/zenodo.10798075
OpenGeoSys 5 (Coupled Thermo-Hydraulic-Mechanical-Chemical Processes Finite Element Method Code)	Kolditz ^45^	https://www.opengeosys.org/ https://doi.org/10.1007/s12665-012-1546-x
Spatial continuity analysis code	Pyrcz ^41^.	https://pypi.org/project/geostatspy/ https://doi.org/10.5281/zenodo.12667036

### Method details

In this section, we detail the acquisition and transformation of the aperture field data from a fracture surface and the subsequent analysis to understand its spatial continuity. This analysis forms the basis for creating a finite element method (FEM) coupled hydro-mechanical numerical model.

This study utilised a sample of gneiss from Freiberg, Germany. Sourced from the first mining level at approximately 125 m depth from the Freiberg Gneiss dome, it is a 540 Ma old metamorphic altered grano-diorite. Petrographic analysis indicates a composition predominantly of plagioclase (41%), quartz (38%), and biotite (21%), with a median grain size of 0.519 mm, 0.505 mm, and 0.762 mm. A 15cm core was extracted and fractured, then each fracture surface was scanned using a laser profilometer.

**Figure 10:**
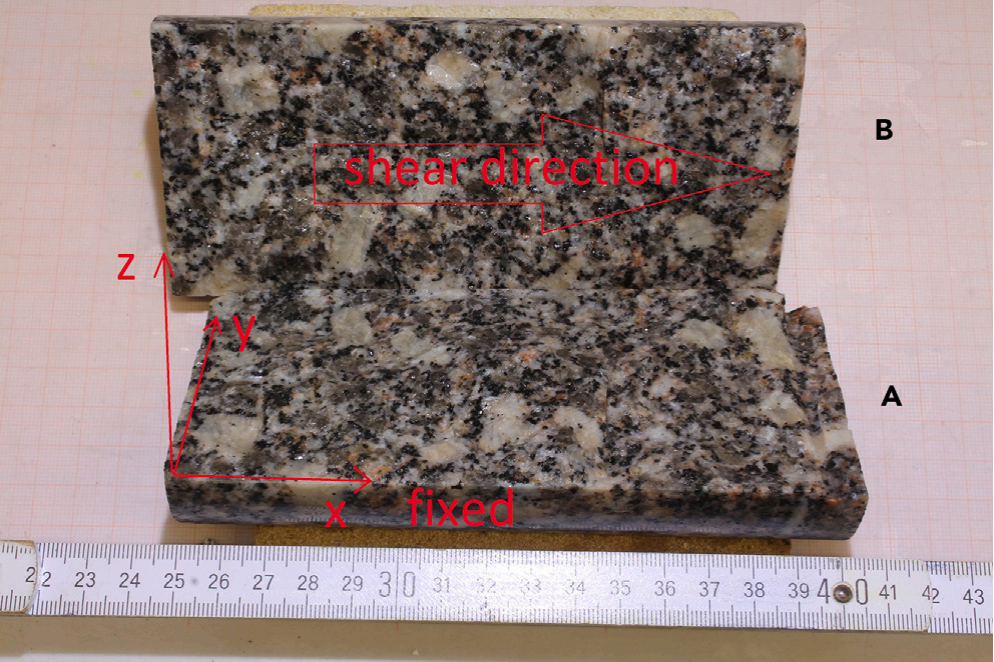
Freiberg gneiss sample whose fracture scans of bottom surface (A) and top surface (B) are used for the aperture field calculation and modeling.

**Figure 11:**
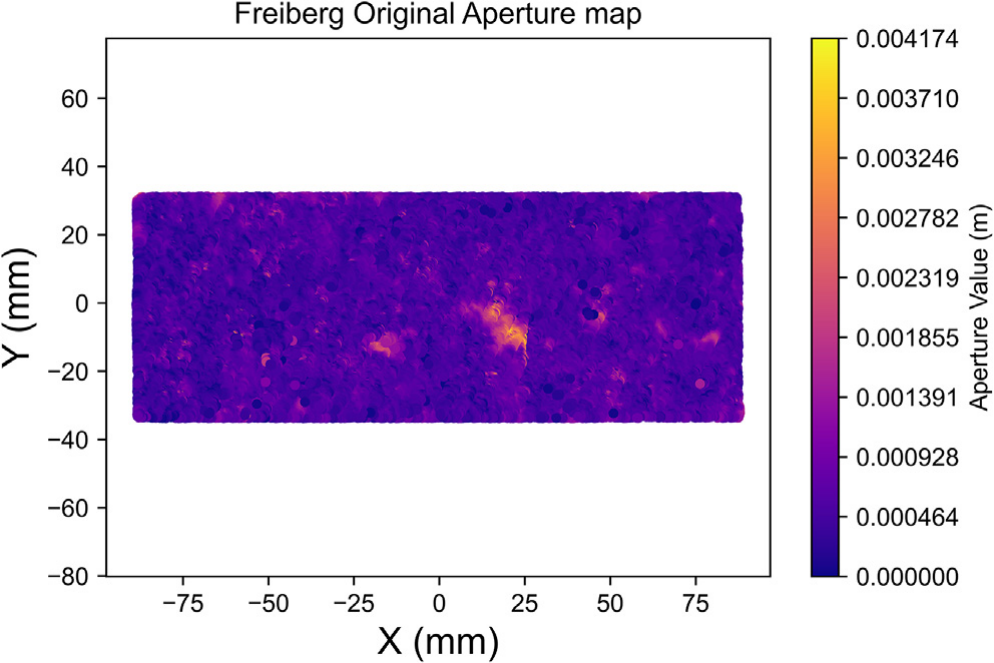
Freiberg gneiss fracture aperture field derived from the difference between the top and bottom fracture’s rough surfaces.

Scans of both fracture surfaces were imported into *Tecplot* for alignment, a simulation and visualisation tool particularly useful for numerical methods. The surfaces were then matched whilst ensuring minimal overlap and optimal proximity. The aperture at each XY point (see Figure 10 for the bottom A and top B surfaces and their coordinate system reference) was determined by editing PyVista’s^53^ 2D linear Delaunay triangulation for one fracture surface, - a Python visualisation and analysis library for geological data – to allow projecting vertical lines from points on the opposing surface until intersecting the triangulated surface, thereby calculating the aperture.^52^ A rendering of the aperture map produced via the described method is plotted in Figure 11. The aperture field point cloud is unstructured with a mean minimum distance (resolution) of 0.0125 m, or 1.25 mm.

The aperture field was simplified by focusing on the middle portion for computational efficiency (Figure 4). The spatial continuity analysis was performed on this middle portion of the fracture. The same data was used in both the averaging and kriging upscaling processes for the creation of all the models.

#### Data transformation

In preparing the data for geostatistical analysis, particularly for the application of kriging algorithms, it is essential to address the distribution of the dataset. Our initial analysis, as can be seen from the histograms showing data distribution in the Supplementary Material (Figures S8 and S9), reveals the aperture data to exhibit an overall normal distribution, although mildly skewed toward lower values and a pronounced tail toward higher values. Such skewness in the data can potentially complicate analysis, as the kriging algorithm assumes the data are best fitted by a normal distribution to function optimally.

To align our data with these requirements, we employed a normal score transformation, a widely recommended practice for normalising data distributions in geostatistical analysis.^41^^,^^48^^,^^54^ This technique involves a two-step process where each data point is first mapped to its corresponding quantile in a uniform distribution, and then to a quantile in a normal distribution, defined by a mean of 0 and standard deviation of 1, which equates to variance of 1 hence the sill is also set to 1. This allows for more easily identify the range of a semi-variogram model. Essentially, this quantile transformation aims to match each *p-quantile* of the original distribution of the dataset to the *p-quantile* of the target normal distribution, thus preserving the rank order of data points. This process ensures that, post-transformation, the relative hierarchy of data values (from lowest to highest) remains unchanged, while the distribution itself is normalised.

The described process is not only critical for the accurate application of kriging, but also serves to reduce the undue influence of extreme values on the analysis, thus making the dataset more amenable to statistical modeling and interpretation. For a more detailed explanation of this transformation process and its significance in spatial data analysis, we refer to foundational text in the fields.^39^^,^^41^^,^^48^

By assuming the finite variance of aperture values – a reasonable assumption given the geological context and the nature of the data – the variance observed in our dataset is considered representative of the true variance of the model. Therefore, the normal score transformation is applied, allowing us to proceed with further geostatistical analysis on a solid, methodologically sound basis.

#### De-trending or drift removal

A trend is a smooth but systematic, non-random variation across space,^37^ detectable by a semi-variogram failure to reach a stable sill, thus indicating non-stationarity. A trend is the structural component referred to in section 2, which masks the true variability of the random processes. Ergo, it requires removal before spatial continuity analysis. The presence of a trend can be easily verified by the absence of a stable sill in a semi-variogram.^43^ Once Identified, the most common approach to remove the trend is to model it first. Then, the value of the trend is subtracted from the value of the original aperture at the same *XY* locations. This results in the de-trended or residual points.

#### Upscaling

Following the spatial continuity analysis, we established a methodology for predicting aperture for the middle portion of the fracture surface for each element in our numerical model that makes use of the data’s spatial correlation. To evaluate two distinct upscaling approaches, the traditional averaging, and our original methods, we implemented.

(1) A model that employs arithmetic averaging to upscale aperture data within the mesh elements; and
(2) A model that leverages ordinary kriging, informed by our spatial continuity analysis.

The upscaling is facilitated by a graphical user interface^51^ taking the mesh of the numerical model and the input data points. These upscaled values simplify the application of the parallel plate model^20^ on a local scale (local cubic law)^9^^,^^17^^,^^55^ to compute the conductivity of each element. The parallel plate model (commonly known as cubic law) provides an approximation of permeability from the aperture, assuming laminar flow.

**Figure 12:**
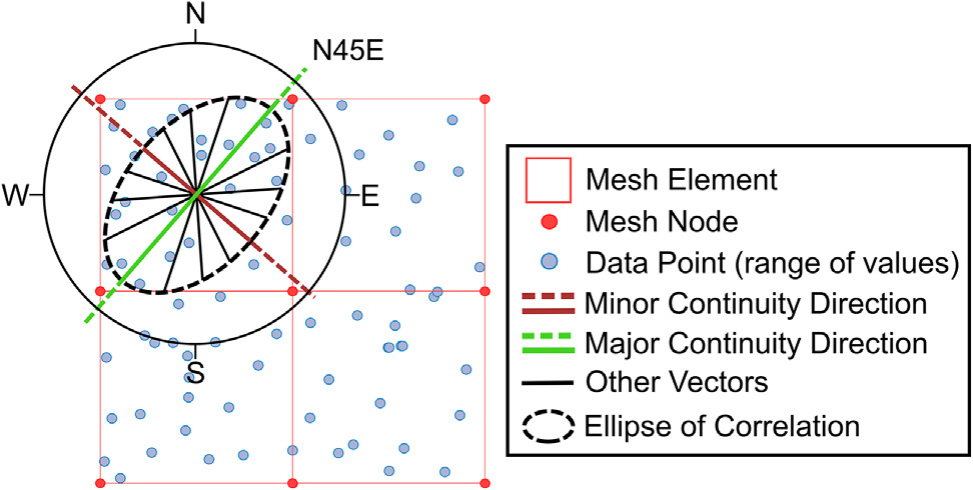
An ellipse of correlation is fitted around the ranges of the experimental semi-variograms calculated for the various directions. The major and minor continuity directions are highlighted in green and red, respectively. These are projected (dashed lines) to allow an easier reading of their orientations with respect the coordinate system. The kriging algorithm is used to predict the value at the center of the mesh’s element through the correlation ellipse, which uses only the points that are statistically correlated to each other. The code to upscale a property using its spatial continuity information through kriging and mapping it onto a numerical method’s mesh is available.

For the kriging-based model, a correlation ellipse – oriented according to the major and minor continuity vectors identified in our analysis – is placed at the center of each element (Figure 12).

The size and orientation of this ellipse will dictate the calculation of the kriged values, determining the inclusion of data points based on their spatial correlation with the center of the element. It is crucial to note that Figure 12 is for illustrative purposes only; the actual vectors for the middle section of the Freiberg surface aperture may vary in orientation and magnitude.

Given the extensive number of data points (in the order of 100,000), preliminary upscaling was necessary to manage 16GB RAM computer memory on an Intel Core i5-8365U 8^th^ Gen processor system constraints encountered during kriging. The same computer system was used for all calculations. This process involved overlaying a grid of square bounding boxes (BBs) or cells across the dataset. The initial scale sets BB side lengths to match the average minimum point-to-point distance, doubling with each subsequent scale until further subdivision is impractical. Averaging the data within each BB allows for efficient kriging based on established spatial continuity parameters and semi-variogram models, ensuring accurate prediction at mesh element centers. The averaging process is almost instantaneous whereas the kriging method takes several minutes, given the size of the dataset. This fact could be related^56^ with the kriging tool we used (PyKrige)^56^: The kriging algorithm creates a global matrix of all points and assign zero weights to the points outside of the spatial continuity ellipse. This could be optimised by creating a kriging algorithm that creates a smaller matrix to include only the points within the correlation ellipse, reducing the size of the matrix and thus the computation power and time.

### Quantification and statistical analysis

Scans of both fracture surfaces were imported into *Tecplot* for alignment, a simulation and visualisation tool particularly useful for numerical methods. The aperture was determined by editing PyVista’s^53^ 2D linear Delaunay triangulation for one fracture surface, - a Python visualisation and analysis library for geological data – to allow projecting vertical lines from points on the opposing surface until intersecting the triangulated surface, thereby calculating the aperture.^52^

The spatial continuity analysis performed on the aperture field was accomplished using GeoStatsPy toolkit, mentioned in the key resources table.

*Tecplot* files of the numerical models were created from OpenGeoSys (see key resources table) and visualised in *Tecplot*.

Data manipulation, statistical analysis, visualisation and plotting was performed in Python.
