# Supplementary material for: Advancements in coupled processes numerical models: Upscaling aperture fields using spatial continuity
Source: iScience. 2024 Oct 2;27(11):111094. doi: 10.1016/j.isci.2024.111094 (PMC11550576; doi:10.1016/j.isci.2024.111094)
Supplement: Document S1. Figures S1–S9 [file mmc1.pdf]

iScience, Volume *27*

Supplemental information

Advancements in coupled processes numerical models: Upscaling aperture fields using spatial continuity

Gonçalo Benitez Cunha, Christopher Ian McDermott, Alexander Bond, Andrew Fraser-Harris, and Roberto Emanuele Rizzo

Supplementary Material Figures

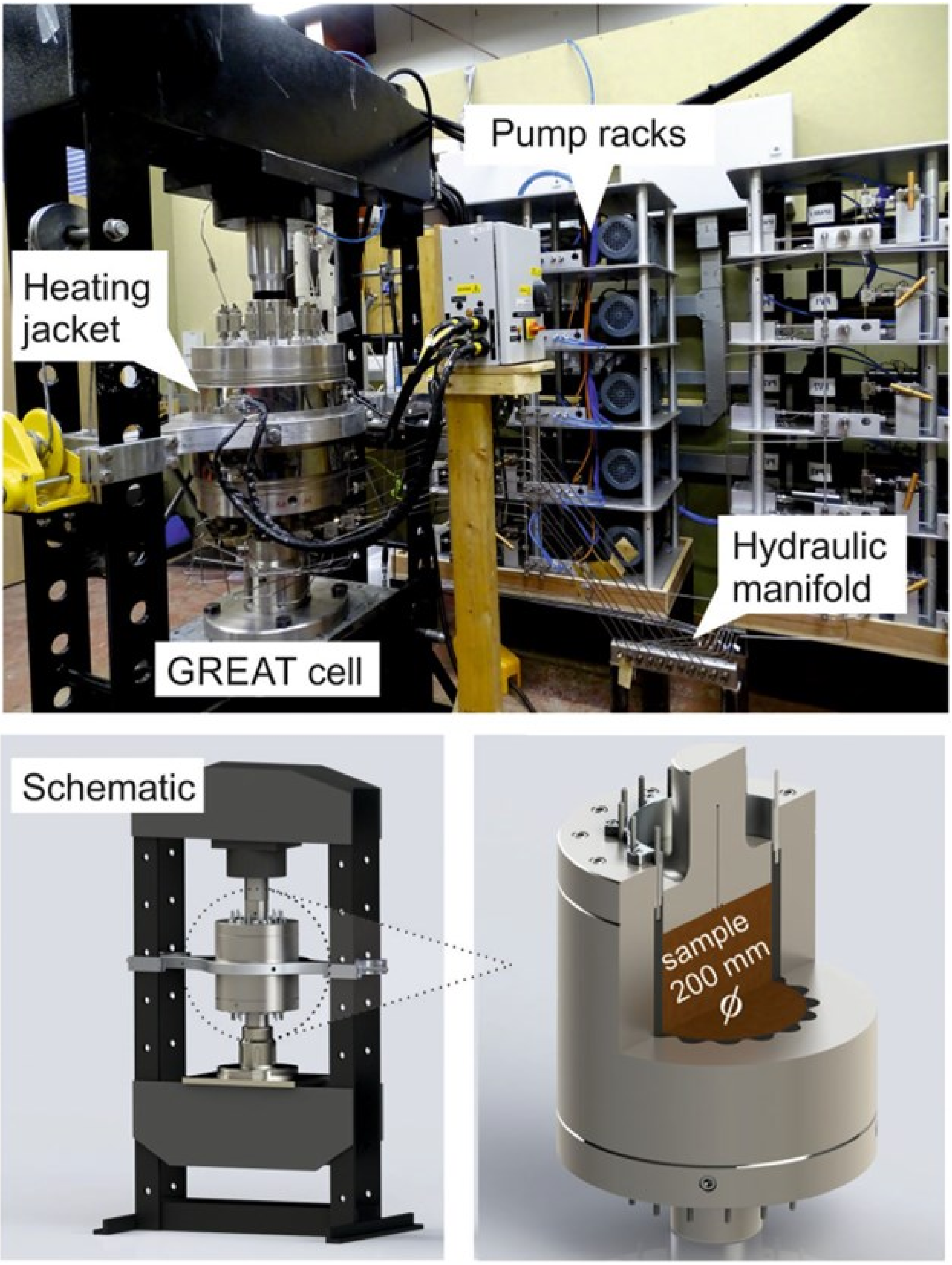

Figure SM 1 – GREAT cell experimental apparatus [11], related to the link between the experimental setup and the model approach in section 3.

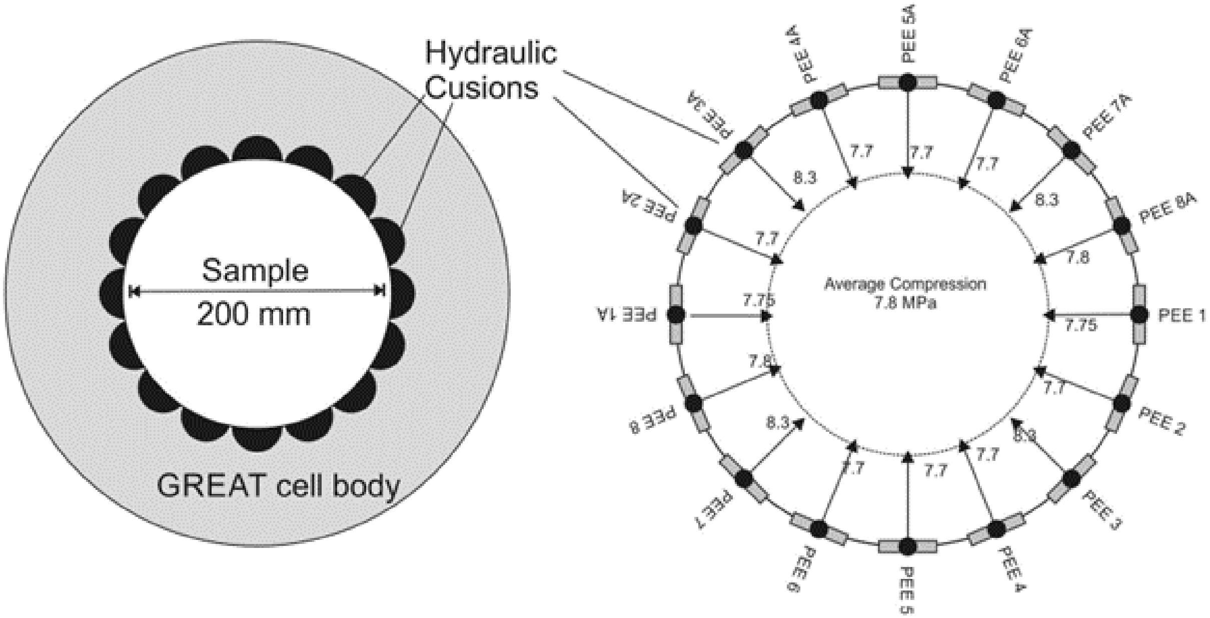

Figure SM 2 – Conceptual diagram representing the hydraulic cushions (Pressure Exerting Elements or PEE) exerting radially controlled stress field on a sample with diameter 194mm. PEE labelling refer only to experimental notation, numbers with arrows refer to fluid pressure in PEE during a particular experiment given as illustration [11]. Related to the link between the experimental setup and the model approach in section 3.

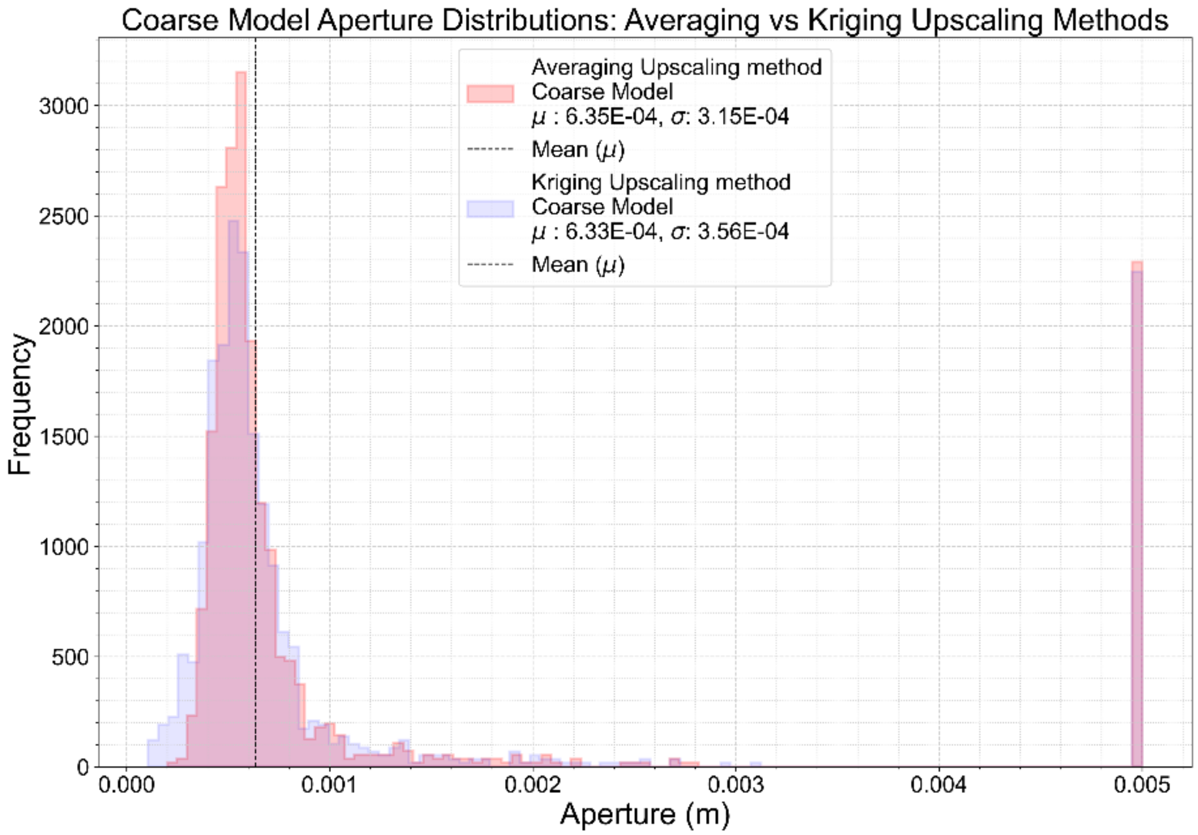

Figure SM 3 – Coarse models aperture distributions. Pink distribution corresponds to the model upscaled using averaging. Blue distribution corresponds to the model upscaled using kriging. The bimodal feature (peak at 0.005m) corresponds to the high aperture fringes added to either side of the models for a better fluid distribution. The means and standard deviations do not include the fringes artificial data. The model upscaled using kriging presents a larger spread than the model upscaled

using averaging. The latter has therefore a higher number of high values of aperture which tend to be more similar to each other. Related to Figure 11 and Figure 13.

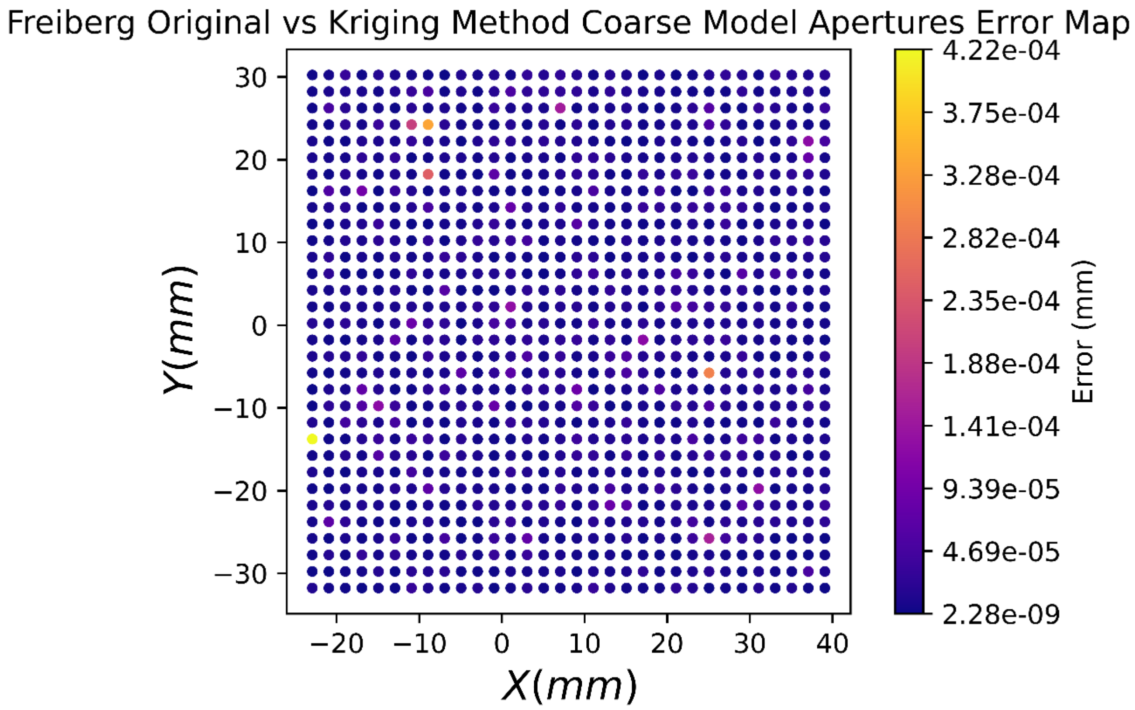

Figure SM 4 – Freiberg fracture original apertures vs kriging method coarse model apertures error map. Related to Figure 13.

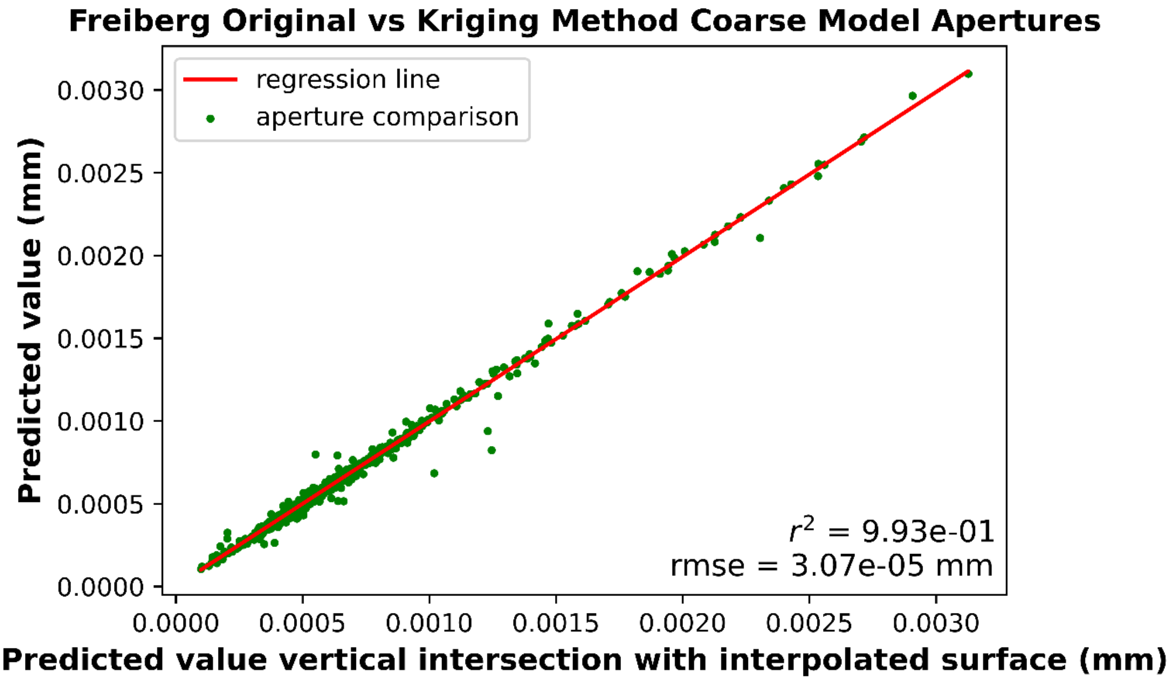

Figure SM 5 – Freiberg fracture original apertures vs kriging method coarse model apertures error regression line. Coefficient of determination (r^2) and root mean square error (RMSE) values are added for reference of the method quality. Related to Figure 13.

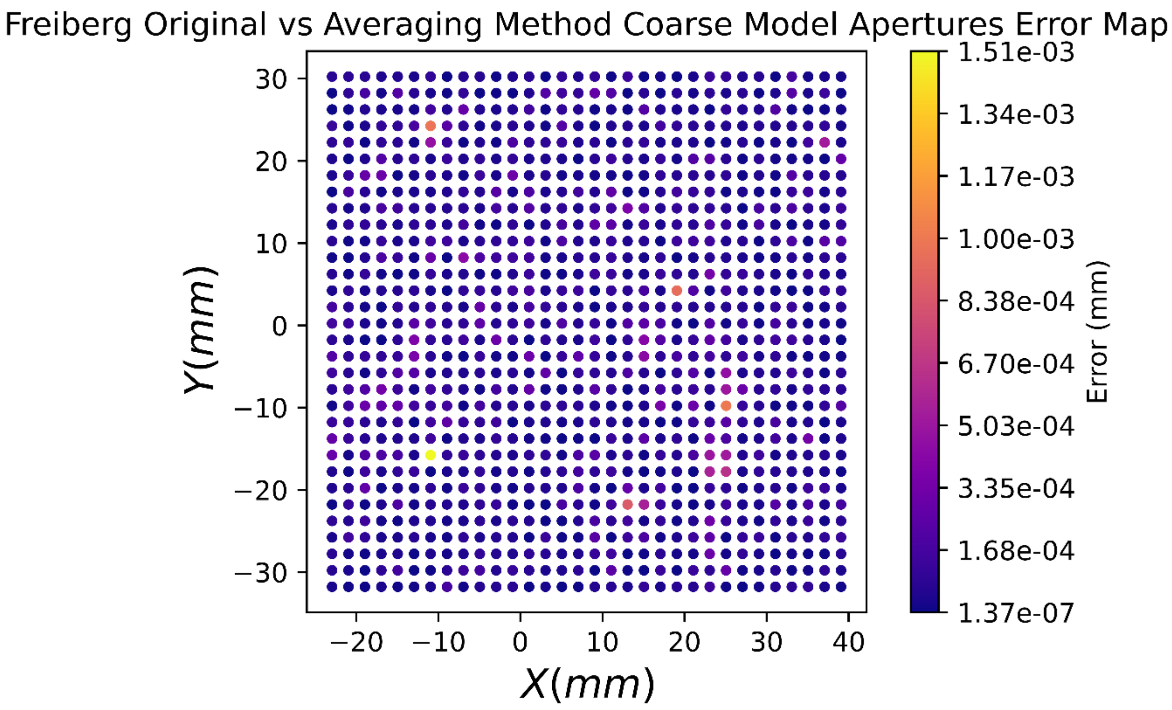

Figure SM 6 – Freiberg fracture original apertures vs averaging method coarse model apertures error map. Related to Figure 13.

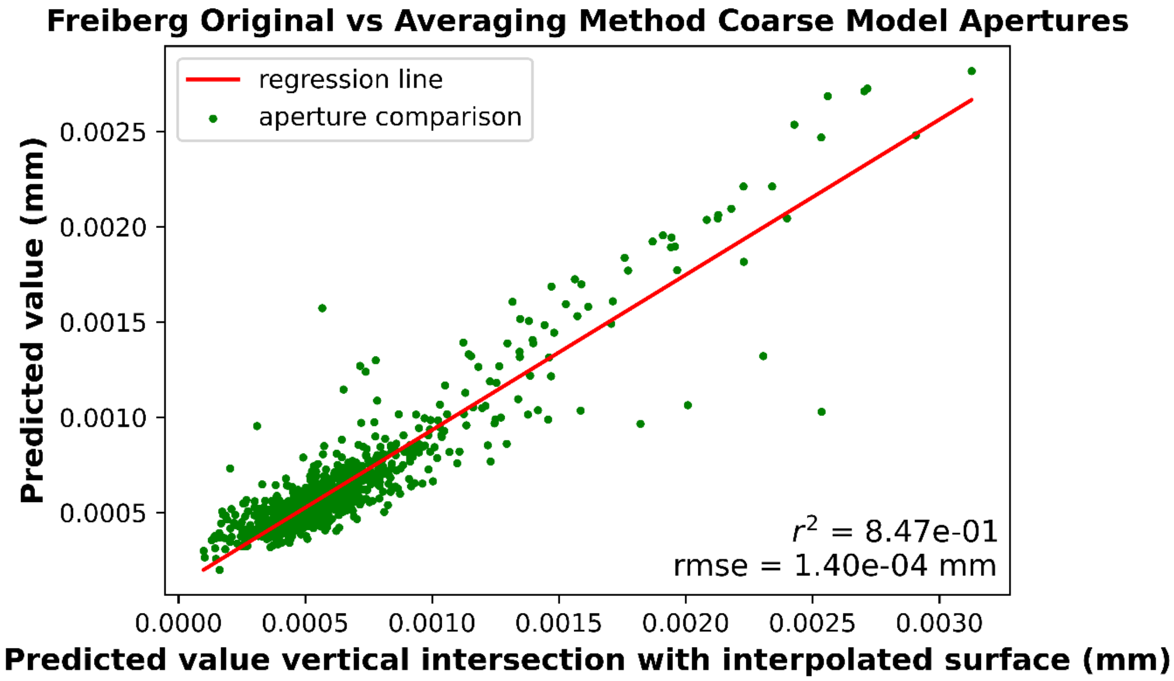

Figure SM 7 – Freiberg fracture original apertures vs averaging method coarse model apertures error regression line. Coefficient of determination (r^2) and root mean square error (RMSE) values are added for reference of the method quality. Related to Figure 13.

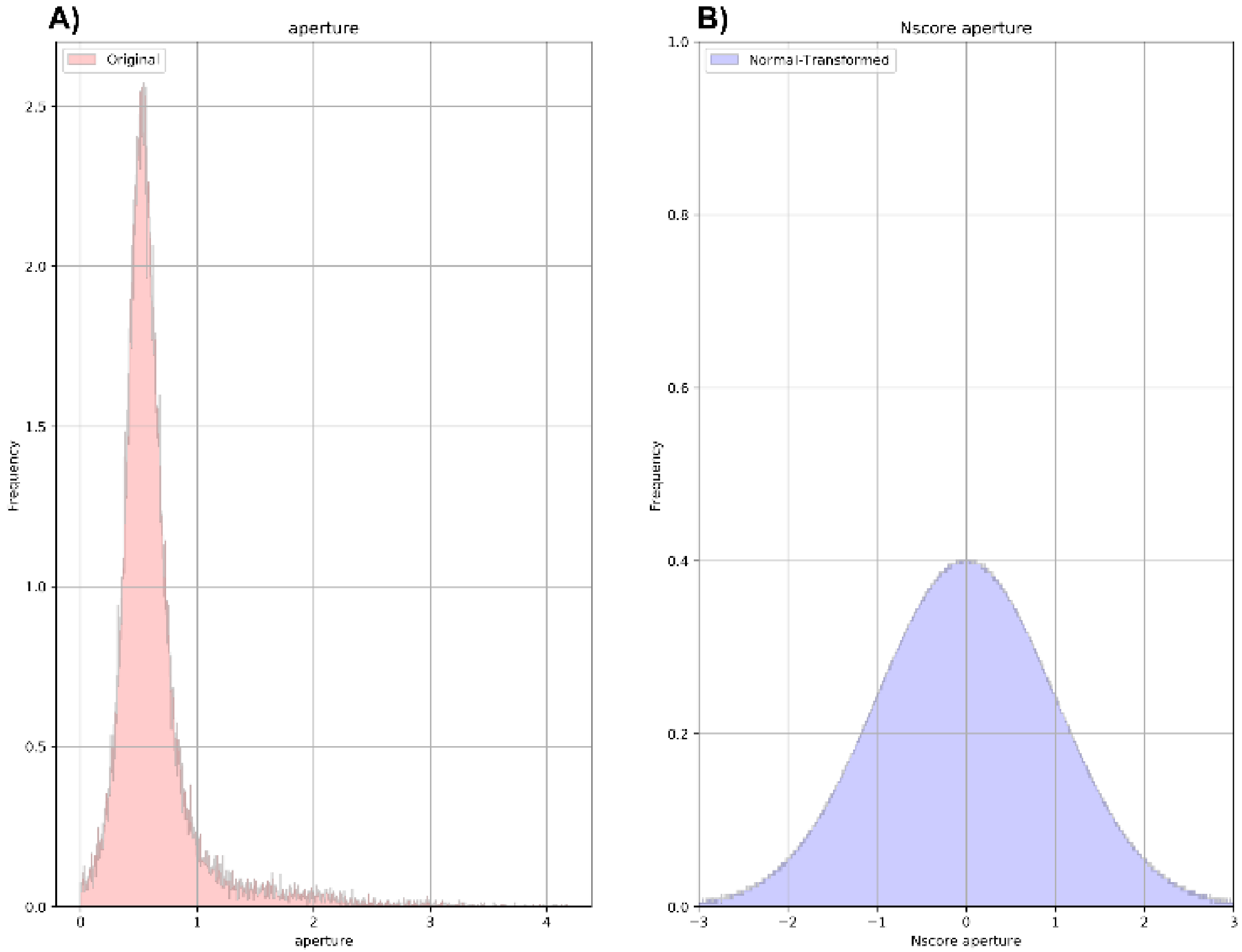

Figure SM 8 - Raw (pink) and normally transformed (purple) probability density function of the central portion aperture data. Related to “Data Transformation” sub-section in “Method details” section.

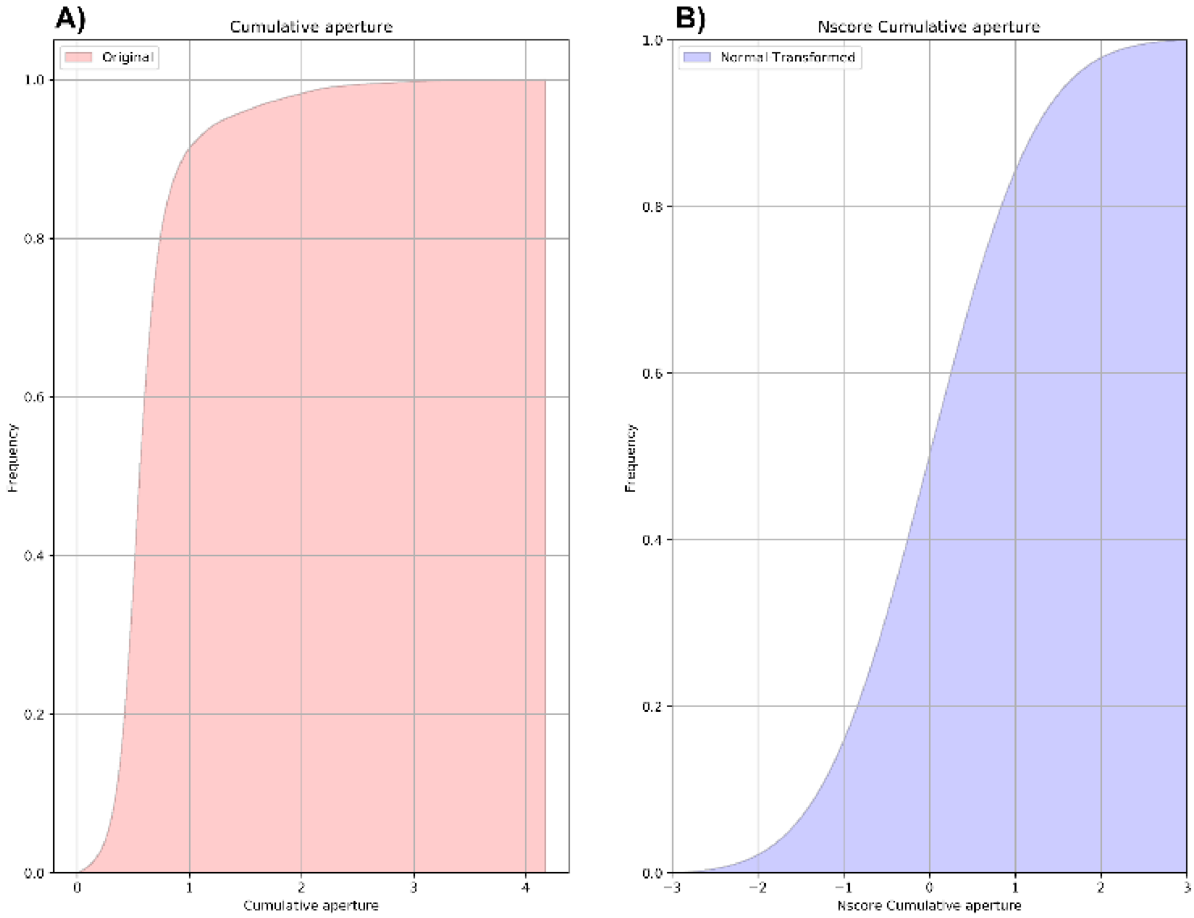

Figure SM 9 - Cumulative density function of raw (pink) and normally transformed (purple) of the central portion aperture data. Related to "Data Transformation" sub-section in "Method details" section.
